# Predicting bentonite swelling pressure: optimized XGBoost versus neural networks

**DOI:** 10.1038/s41598-024-68038-x

**Published:** 2024-07-26

**Authors:** Utkarsh, Pradeep Kumar Jain

**Affiliations:** Department of Civil Engineering, Maulana Azad National Institute of Technology, Bhopal, 462003 India

**Keywords:** Xtreme gradient boosting, Swelling pressure, Soft soil, Bentonite, Optimization, Civil engineering, Solid Earth sciences

## Abstract

The swelling pressure of bentonite and bentonite mixtures is critical in designing barrier systems for deep geological radioactive waste repositories. Accurately predicting the maximum swelling pressure is essential for ensuring these systems' long-term stability and sealing characteristics. In this study, we developed a constrained machine learning model based on the extreme gradient boosting (XGBoost) algorithm tuned with grey wolf optimization (GWO) to determine the maximum swelling pressure of bentonite and bentonite mixtures. A dataset containing 305 experimental data points was compiled, including relevant soil properties such as montmorillonite content, liquid limit, plastic limit, plasticity index, initial water content, and soil dry density. The GWO-XGBoost model, incorporating a penalty term in the loss function, achieved an R^2^ value of 0.9832 and an RMSE of 0.5248 MPa in the testing phase, outperforming feed-forward and cascade-forward neural network models. The feature importance analysis revealed that dry density and montmorillonite content were the most influential factors in predicting maximum swelling pressure. While the developed model demonstrates high accuracy and reliability, it may have limitations in capturing extreme values due to the complex nature of bentonite swelling behavior. The proposed approach provides a valuable tool for predicting the maximum swelling pressure of bentonite-based materials under various conditions, supporting the design and analysis of effective barrier systems in geotechnical engineering applications.

## Introduction

Bentonite, a naturally occurring clay mineral predominantly composed of montmorillonite, exhibits remarkable swelling characteristics that have garnered significant attention across various industrial applications^1,2^. The unique property of bentonite One of its most important functions is its ability to expand by absorbing water and electrolytes inside of its layered structure^3^. This swelling pressure is the term for the physical stretching of the bentonite that is resisted by the tight confinement condition introduced above^3^. Bentonite's employment as a buffer material in deep geological repositories for radioactive waste disposal is an important use for the material. Its poor hydraulic conductivity, which prevents it from being leached from the system, and its strong swelling pressure to the extent that it has to be secured against outside pressure play a fundamental role in the protective function of its structure^4,5^.

Bentonite has found various applications in the petroleum engineering domain beyond its use in drilling fluids. Towler and Ehlers^6^ investigated the potential use of hydrated bentonite in plugging oil and gas wells, proposing a theory to predict the pressure that could be withstood by a hydrated bentonite plug. Englehardt et al.^7^ successfully used compressed sodium bentonite nodules to plug and abandon wells in California. In enhanced oil recovery (EOR) applications, thermosensitive water-soluble polymers incorporating bentonite have been developed to address challenges associated with high-temperature and high-salt reservoirs^8,9^. Additionally, bentonite has been explored for the treatment of oilfield wastewater^10^. These diverse applications demonstrate the versatility and potential of bentonite in various aspects of petroleum engineering.

Novel though these functions be, the swelling of bentonite remains a big challenge, especially with changes like the environment or moisture content and consolidation levels which act as a major factor in its swelling capabilities, and have a direct impact on the number of industrial applications^3^. For example, bentonite's strong adsorption and retention ability makes geosynthetic clay liners (GCLs), which have been utilized for environmental containment, and efficient pollution barriers^11^. While that is, in the other cases, considerable volume swelling could adversely affect the integrity and transport of the confined medium in these liners^11^. Additionally, some problems related to increasing the amount of unblended bentonite in pellet preparation during the water-soil mixing led to uneven wetting. This is not an easy job to do because the bentonite acts as a barrier to redistribute the oil contained inside. As a potential stuffing material which may conceivably block the effectiveness of the sealing. Moreover, it might promote thus causing structural and economic fragility and instability in the system as a whole^3^. Nevertheless, as the components that come into play are so intricate, the expectation to isolate their relationship to individual events becomes even more difficult and impossible to predict in advance bentonite's behavior while it is swelling^12^. To achieve efficiency and at the same time reduce expenses and environmental impacts, such balance should be applied,this includes inherent properties of grass-based systems and application-specific factors^3^. However, because of the complicated interactions between several contributing elements, it is difficult to anticipate the behavior of bentonite swelling^12^.

The amount of montmorillonite, which has a major influence on the clay's capacity to soak up water and enlarge, is one of the major elements determining the swelling behavior of bentonite^13,14^. The classic strength of the ambient surroundings determines if the tight bonding will form between the tiny strata layers or if electro-interactions will prevail (Benayoun et al.)^15–17^. Moreover, the water that is present initially helps to impede imbibition, and consequently, lower swelling pressure is observed due to a higher moisture content ^3^. However, on the other hand, although it is certainly the case that the connection between bentonite and specific surface area properties is very strong, the bond between bentonite and swelling characteristics is as well as strong. The bond is an example where a higher volume of water might get soaked up because the surface area seems to be greater. Due to this, the reaction expansion of concrete that was seen is more pronounced^18^.

Minerals more stone-like ones like carbonates, feldspars, micas, and quartz, relative to those which swell, are more effective in their effort to prevent swelling. By taking up space inside the clay matrix, these minerals dilute the amount of montmorillonite and reduce the bentonite mixture's overall swelling capacity, changing its structural and chemical characteristics^3,19^. However, till now the main factor of bentonite swell pressure has been viewed as dry density. Because the particles are packed closer together at a greater dry density, the swelling pressure is usually higher as well, improving the clay's resistance to expanding in volume during confinement^20^. In the meantime, the clay consistency and plasticity (determined by the Atterberg limits) play a huge role in the bentonite behavior during the simulation of different conditions. Such characteristics as moisture content at the transition state to power bentonite up or down are also crucial for how this sediment functions^3^.

With the enormous amount of time, there exist several empirical^21,22^, and semi-empirical formulae that were developed to predict the bentonite swelling and bentonite mixes pressure (Tripathy et al. 2014). Nevertheless, because these models are customized for certain kinds of bentonite or mixes, they frequently cannot be used in various situations^3,23^. As a result, there is an urgent need for more sophisticated forecasting techniques that can precisely combine the important influencing variables and provide trustworthy forecasts for various applications. Swelling pressure is the soil’s quality continually improving for several decades. The latest computational methods in machine learning can accurately predict it^20,24, 25^. The unique properties of bentonite, such as its complicated interactions with environmental conditions and variable swelling behavior, provide hurdles to the efficacy of current prediction models.

Recent advancements in artificial intelligence, machine learning, and deep learning have shown promise in accurately predicting various properties across different domains, including healthcare^26^, petroleum engineering^27,28^, and computer vision^29^. These techniques have the potential to revolutionize the prediction of complex phenomena, such as bentonite swelling pressure. In population genetics, deep learning approaches have gained popularity, facilitated by the advent of massive genomic datasets, powerful computational hardware, and complex architectures. These methods have been used to identify population structure, infer demographic history, and investigate natural selection^30^. Despite the impressive performance of these techniques, several challenges remain, such as the need for large amounts of training data, computational resources, and interpretability^26–30^. Addressing these challenges and harnessing the power of computational methods will be crucial for advancing various fields, including the prediction of bentonite swelling pressure.

Despite the growing application of machine learning techniques in predicting the swelling pressure of bentonite, there remains a lack of comprehensive studies that compare the performance of different models and address the specific challenges associated with this task. Many existing studies focus on developing a single predictor for swelling pressure without thoroughly exploring the limitations of current approaches or clearly articulating the novelty of their proposed methods^31^. Moreover, the input features used in these studies are often limited, failing to capture the complex relationships between soil properties and swelling behavior (Tinoco et al.^16^). Furthermore, detailed discussions on the implementation of machine learning models, particularly in terms of reproducibility and automation, are often neglected in the current literature^32^. In this study, we aim to bridge these gaps by conducting a comparative analysis of various machine learning models, including CNN and LSTM, and by incorporating a diverse set of input features that encompass the key factors influencing the swelling pressure of bentonite. We also provide detailed information on the implementation of our models to ensure reproducibility and facilitate future research. Our research contributes to the field by providing a comprehensive evaluation of different modelling approaches and by developing a robust framework in-respect to estimate the swelling pressure of bentonite under variety of conditions. The insights gained from this study can guide future research efforts and support the development of more accurate and reliable models for predicting the behavior of bentonite-based materials in geotechnical engineering applications.

Related research in geotechnical engineering, rock mechanics, and machine learning has been conducted to address similar challenges. Machine learning techniques like Artificial Neural Networks (ANNs), Support Vector Machines (SVMs), and Random Forests (RFs) have been successfully applied to predict rock strength parameters^33^ and model complex relationships in slope stability analysis^34^. Optimization algorithms such as Genetic Algorithm (GA) and Pattern Search (PS) have been used to improve the performance of fuzzy inference systems for predicting roof fall rates in underground coal mines^35^. Deep learning models have been employed to estimate the brittleness of deep shales by integrating various geological and geotechnical parameters^36^. Image processing and deep learning techniques have been utilized for analyzing and classifying dust pollution in mining processes^37^. Ultrasonic testing and machine learning algorithms have been applied to determine crack stress thresholds in rocks^38^, and hybrid machine learning models have been used for estimating particulate matter concentration in coal mines^39^.

Despite these advancements, there are still limitations and gaps in the existing research that need to be addressed. To bridge this technological gap, the current work presents a unique machine learning strategy that uses the ADAM optimizer in conjunction with three neural network models: RBFNN: Radial Basis Function Network, LSTM-NN: Long Short-Term Memory Neural Network, and CNN: Convolutional Neural Network. Furthermore, two optimization methods are used to develop and fine-tune the extreme gradient boosting (XGBoost) algorithm: The selection of Particle Swarm Optimization (PSO) and grey annual sunshine hours brings more system efficiency and reliability. Comparing the performance of the proposed neural network models and the XGBoost algorithm with the experimental data helps to determine the best technique for an accurate prediction of BB and BB mixtures' optimum swelling pressure. The model quickens working with less precise values and is recommended as a complementary tool for the traditional outcomes, therefore giving a more reliable and usable tool that estimates the swelling pressure needed.

To bridge this technological gap, the current work presents a unique machine learning strategy that uses the ADAM optimizer in conjunction with three neural network models: RBFNN: Radial Basis Function Network, LSTM-NN: Long Short-Term Memory Neural Network, and CNN: Convolutional Neural Network. Furthermore, two optimization methods are used to develop and fine-tune the extreme gradient boosting (XGBoost) algorithm: The selection of Particle Swarm Optimization (PSO) and grey annual sunshine hours brings more system efficiency and reliability. Comparing the performance of the proposed neural network models and the XGBoost algorithm with the experimental data helps to determine the best technique for an accurate prediction of BB and BB mixtures' optimum swelling pressure. The model quickens working with less precise values and is recommended as a complementary tool for the traditional outcomes, therefore giving a more reliable and usable tool that estimates the swelling pressure needed.

## Data

There are 305 experimental data points from various published sources^3^ that investigate the maximum swelling pressure of BB mixtures that are proven as a credible source. This collection contains a range of bentonite varieties, regional blends, and techniques for determining the optimum swelling pressure. This approach implies an adequate and open reflection on the matter to be addressed. The data presented in Table 1 highlights the significant variability observed in the properties of BB mixtures across various studies^12,18,20,40–50^.

**Table 1:** Range of parameters for bentonite and bentonite mixtures from various studies.

Source	W_L_/%	${\sigma }_{max}^{Sw}$/MPa	W_P_/%	W_i_/%	M/%	I_P_/%	γ_d_/g cm^−3^
Börgesson et al.^41^*	400–400	1.41–14.64	70–70	11–11	75–75	330–330	1.43–1.78
Liu et al.^46^	241–241	0.37–10.37	52–52	8–16.8	30.97–65.39	205.8–205.8	1.4–2
Bag and Jadda^20^	172–230	0.52–9.41	43–52	8–16	41–63	120–187	1.4–1.8
Komine et al.^45^	128.7–565	0.33–10.82	23.7–47.2	8.3–18.15	57–84	90.3–517.8	1.25–1.89
Zeng et al.^50^	176.9–358.1	0.13–5.23	30.6–39.4	7.7–9.8	25.8–60.2	146.3–318.7	1.27–1.99
Sun et al.^48^	229–229	1.20–11.23	65–65	10–10	60–60	164–164	1.21–1.83
Komine and Ogata^18^	473.9–473.9	0.45–2.67	26.6–26.6	5.95–16.5	48–48	447.3–447.3	1.43–2.01
Baille et al.^40^	178–178	0.14–7.87	56–56	9.5–9.5	60–60	122–122	1.83–1.21
Schanz and Tripathy^47^	178–178	0.07–9.21	56.1–56.1	9.9–9.9	80–80	121.9–121.9	1.10–1.72
Kahr et al.^44^*	140–140	1.71–39.39	50–50	9–9	66–66	90–90	1.39–1.90
Gray et al.^43^*	257–257	4.08–18.94	49–49	7.5–7.5	79–79	208–208	1.55–1.77
Schanz and Al-Badran^12^	276–276	0.09–4.23	37–37	11.14–11.14	74.5–74.5	239–239	1.14–1.75
Villar and Lloret^49^	102–102	0.38–16.77	53–53	8.87–27.67	90–90	49–49	1.26–1.76

Facts have been collected from^3^.

BB mixes are used as buffer materials in deep geological radioactive waste repositories. It is crucial to accurately determine the optimum swelling pressure of bentonite since it impacts the durability and sealing properties of the barrier system over the long term^5,51,52^. The complex interconnection of factors such as montmorillonite content, ionic strength, initial moisture content, and specific surface area significantly influence the clay's swelling capacity^14–18^. Furthermore, the most important element affecting bentonite's swelling pressure is its dry density^19^. The nature of bentonite under various conditions is, however, greatly affected by the effect of Atterberg limits such as the Bag and Jadda^20^. Over the past century, several models and equations, both empirical and semi-empirical, have been developed to estimate the swelling pressure of BB mixtures^21,22,53^. In his work, Çimen et al.^23^ underlined that the limitation of generality and the demand to have a different course design for business compositions limit their conduct. In light of these drawbacks, more sophisticated predictive models that incorporate the key variables affecting bentonite's swelling behavior are required to provide an estimate that is more accurate and consistent under various circumstances and applications^54,55^.

The dataset covers a wide range of parameters, including montmorillonite content (M/%), liquid limit (W_L_), plastic limit (W_P_), plasticity index (I_P_), initial water content (W_i_), dry density (γ_d_), and the corresponding maximum swelling pressure (${\sigma }_{max}^{Sw}$). Anyway, there were some cases where the data stored there did not include a proper piece of information about the montmorillonite content. Therefore, our analysis accounted for the structural differences and initial conditions of the material by using M/% estimation via Eq. 1, ensuring a comprehensive study. For instance, the suitability of the model is dependent on the variation of the parameters and test conditions. Therefore, the model fits into a variety of scenarios. With a mean of 4.16 MPa and a standard variation of 4.64 MPa, the greatest swelling pressure, which varies from 0.07 to 39.38 MPa, is very variable, as Table 2 illustrates. This broadness suggests that several things like the type of bentonite, the degree of moisture in the material, and the amount of water will, in essence, have an impact on the swelling of the bentonite.

1 $$M/\% = \frac{B^\circ \times M^\circ /\% }{{100}}$$

where M/% represents the percentage of montmorillonite, B° is the percentage of bentonite in the mixture, and M° /100 represents the percentage of montmorillonite in pure bentonite. Another demonstration of those vast limitations of the sediment type conveyed by the Table 1 is the significant ranges of the content of montmorillonite (25.8–90%), liquid limit (102–565%), plastic limit (23.7–70%), plasticity index (49–517.8%), and dry density (1.10–2.01For a precise estimation of the maximum swelling pressure, it is crucial to comprehend how these qualities interact since they have a direct impact on the swelling characteristics of BB mixes. Data from Table 1 illustrates that there is considerable diversity, as a result of bulk mixtures (BB mixtures), in multiple soil parameters.

**Table 2:** Comprehensive statistical analysis of various bentonite properties covering the whole dataset, focusing the important variability percieved in the compiled data.

Parameter	Units	Std	Min	Mean	Skewness	Max	Kurtosis
γ_d_	g cm^−3^	0.19	1.10	1.59	− 0.13	2.01	− 0.25
W_L_	%	141.87	102	257.65	0.65	565	− 0.86
W_P_	%	11.79	23.7	43.67	− 0.03	70	− 0.95
W_i_	%	5.43	5.95	13.02	1.70	40.4	3.51
Mt.c	%	18.03	25.8	67.46	− 0.31	90	− 0.94
I_P_	%	148.59	49	213.98	0.60	517.8	− 0.95
${\sigma }_{max}^{Sw}$	MPa	4.64	0.07	4.16	3.06	39.38	15.51

### Feature selection and multicollinearity

**Figure 1:**
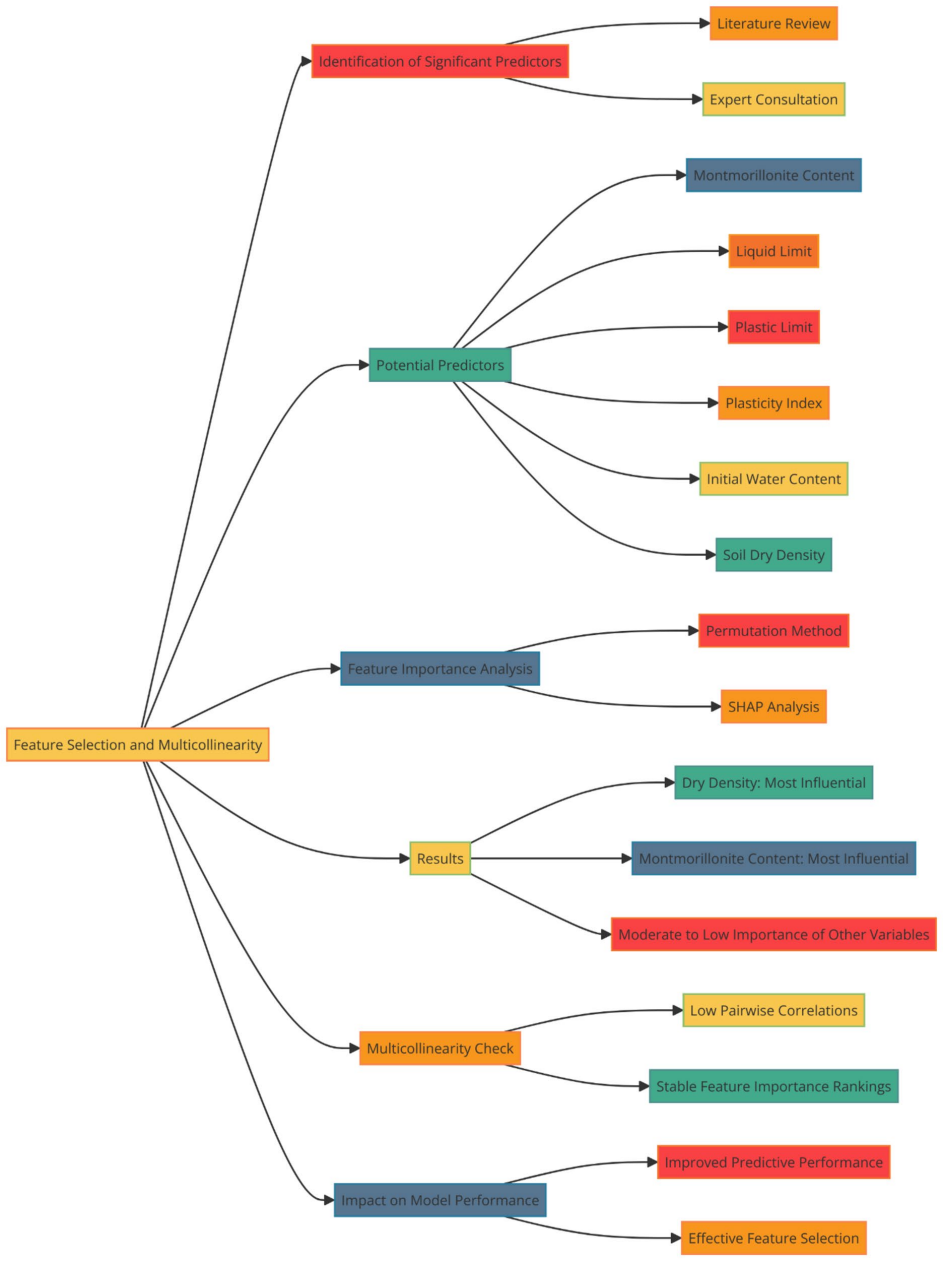
Feature selection and multicollinearity analysis process flowchart.

To identify the most significant predictors of swelling pressure from the dataset, a combination of domain knowledge and statistical techniques was employed. Firstly, based on a thorough literature review and expert consultation, a set of potential predictors, including M/%, W_L_, W_P_, I_P_, W_i_, and γ_d_, was selected. These variables were chosen due to their known influence on the swelling behavior of bentonite and bentonite mixtures. Second, a feature importance analysis was carried out using permutation and SHAP (SHapley Additive exPlanations) approaches in order to evaluate the relative importance of these predictors and pinpoint any possible multicollinearity problems. While the permutation technique shuffles each feature's value at random and tracks the effect on the model's performance, SHAP offers a more thorough analysis by taking into account every potential feature combination. The findings of these studies indicated that the factors with the greatest degree of influence were γ_d_ and M/%, with the remaining variables being of moderate to low significance. Multicollinearity was not found to be a significant issue among the selected features, as indicated by the low pairwise correlations and the stability of the feature importance rankings across different model iterations. The inclusion of these selected features in the machine learning models led to improved predictive performance compared to models using a more limited set of predictors, highlighting the effectiveness of the feature selection process in capturing the key drivers of swelling pressure. A flowchart for the same has been visualized in Fig. 1.

## Methods

### Extreme gradient boosting (XGBoost)

In this regard, the capacity of XGBoost to handle machine learning has been demonstrated and it outperforms in domains such as forecasting and exchange rate prediction^56^. The swelling pressure of bentonite is controlled by a wide range of connected soil variables, and XGBoost can be a useful tool for forecasting this pressure due to its capacity to capture complicated correlations in data.

The XGBoost model works on an algorithm which is an additive tree species which each predicts on their own. Specifically, for a dataset with n examples and m features, D = {(x_i_, y_i_)} (|D| = n, x_i_ ∈ R^m^, y_i_ ∈ R), the XGBoost model uses K additive tree functions to predict the output:

2 $$\hat{{\rm y}}i = \mathop \sum \limits_{k = 1}^{k} f_{k} \left( {x_{i} } \right),\;f_{k} \in F$$

where F = {$f_{{}}$(x) = w_q(x)_} (q: R^m^ → T, w $\in$ R^T^) is the regression tree space. The function q encodes the structure of each tree, mapping examples to leaf indices, and T is the number of leaves in the tree. Every ${f}_{k}$ should be connected to an individual subtree labeled q and a leaf weight w.

To train the ensemble of tree functions, XGBoost minimizes a regularized objective function:

3 $${\text{L}}\left( {\Phi } \right){ } = { }\mathop \sum \limits_{i} l(\hat{{\rm y}}_{i} ,y_{i } ){ } + { }\mathop \sum \limits_{k} {\Omega }\left( {f_{k} } \right)$$

which is a smooth, differentiable convex loss function $l$ that computes L2-norm ŷ_i_ and y_i_. The second term Ω penalizes the complexity of the model, promoting the selection of simple and predictive tree functions. A crucial element of XGBoost method is the first-order approximation of the objective function to optimize it using the additive technique. At each iteration t, a new tree function f_t_ is added to minimize the following approximated objective:

4 $$\text{L}(\text{t}) \simeq \sum_{i=1}^{n}[l({y}_{i},{\widehat{y}}_{i}^{\left(t-1\right)})] +\text{ gi}{f}_{t} (\text{xi}) + \frac{1}{2} \text{hi}{f}_{t}2 (\text{xi})] +\Omega ({f}_{t})$$

where g_i_ and h_i_ are the first and second-order gradient statistics on the loss function. Yet,the current XGB method necessitates the loss function to be convex, and some advancements have been made to relax this particular constraint, making them more generalized^57,58^.These generalized approaches allow for the use of non-convex loss functions, which may better capture the complex swelling behavior of bentonite, especially when the predictor variables exhibit heavy-tailed distributions.

In addition, the authors have also enhanced the Generalized Extreme Gradient Boosting technique such that it can also function effectively for many parameters of the probability distributions^58^.This multi-objective parameter regularized tree boosting method enables the simultaneous modeling of multiple parameters of the predictor variable's probability distribution, potentially leading to improved prediction accuracy of the swelling pressure. The proposed approach can be greatly enhanced by utilizing the all-scale feature of the XGBoost and the wide range of variability presented by the generalized and multi-objective methods whose features are suitable for predicting swelling pressures among bentonite-based materials, in a wide range of conditions and applications. The ability to handle non-convex loss functions and model multiple parameters of the predictor variable distribution can be particularly beneficial in scenarios where the swelling behavior is complex and the underlying probability distributions are not well-captured by traditional methods.

**Figure 2:**
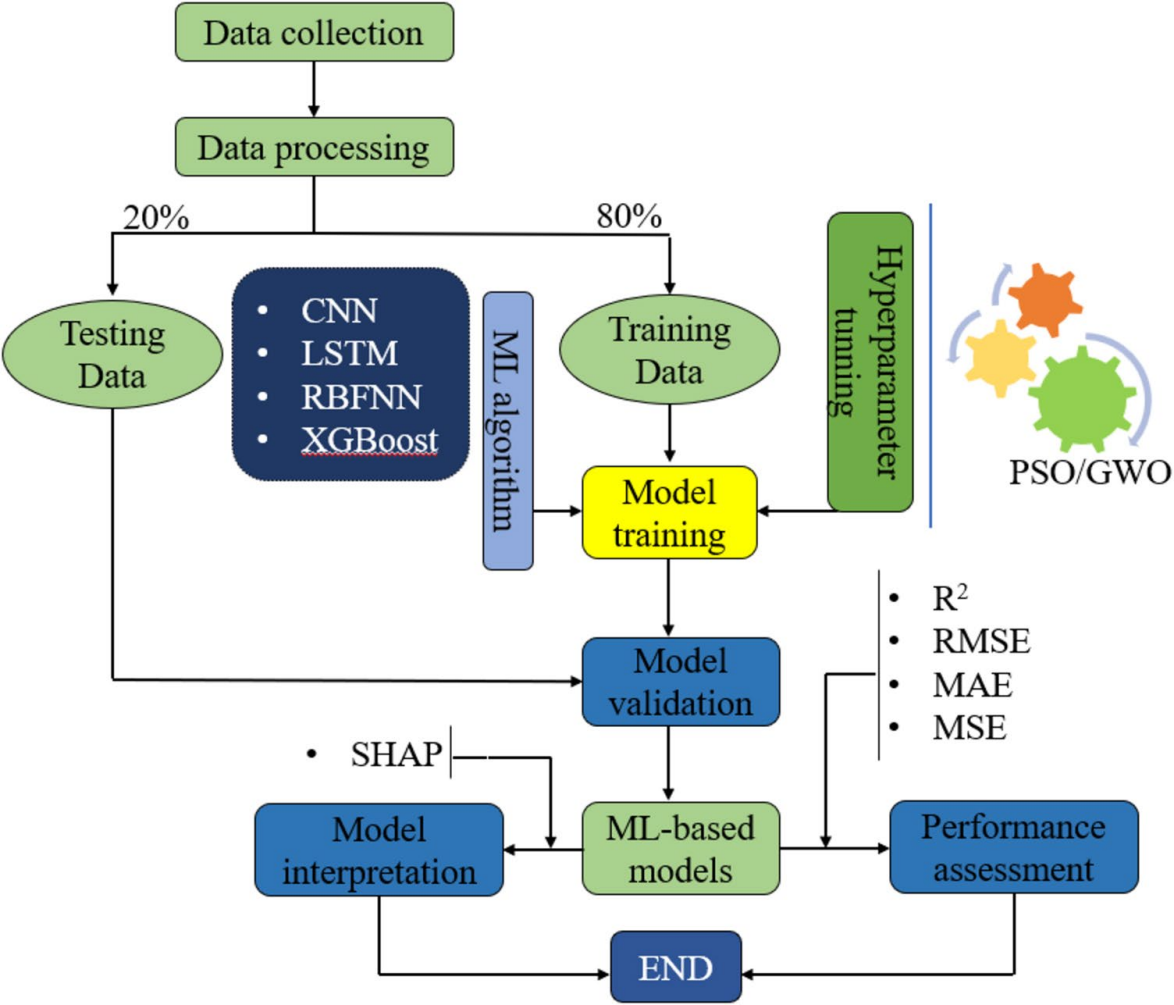
Model Development flowchart.

The procedures utilized for designing machine learning models aimed towards the prediction of the ultimate swelling pressure of bentonite are total featured below in the flowchart of Fig. 2:

### Neural networks

#### Convolutional neural network

Convolutional Neural Networks (CNNs) are a powerful deep learning technique that has shown exceptional performance in various computer vision tasks, including image classification, segmentation, and object detection^59–61^. The capacity of CNNs to capture complex dependencies in data makes them suitable candidates for predicting the overburden pressure of bentonite, which in turn is a function of multiple interrelated soil properties. The CNN model employs a hierarchical architecture consisting of convolutional layers, pooling layers, and fully connected layers to process the input data and generate the desired output. The convolutional layers use a set of learnable filters to the input and gather the information with various scales. The pooling layers then down sample the feature maps, reducing the computational complexity and enabling the network to capture more abstract representations. Ultimately, the layers of perceptron learn intricate non-linear connections between features and the target output which, in our case of swelling pressure estimation, would be the estimated result.

One of the most salient capabilities of CNNs is their ability to automatically extract features from the original input data, consequently lowering the amount of manual feature engineering. This is especially useful under circumstances where restrictions of the bentonite swelling pressure analysis cannot be covered using traditional methods due to the complexity of the interactions between soil properties. To effectively model the swelling behavior of bentonite using CNNs, researchers have proposed several architectural modifications and enhancements. As an example, the article talks about the application of the Savitzky-Golay Filtration and Pyramid Pooling layers in the CNN architecture^62^. The use of Savitzky-Golay Filtering technique for preprocessing purpose with an objective to smooth out the input spectra and to reduce noise is also mentioned.This will focus the attention of the neural network on discovering attributes indicative of the environmental properties targeted.In contrast, the Pyramid Pooling layer divides the feature maps into sections of different sizes and performs pooling operations to extract multi-scale data from the spectra. Consequently, the network scans both local and remote data about bentonite taking into account the given context resulting in a comprehensive scan of the data.

Furthermore, the past research also states the potential benefits of using more generalized CNN methods, such as those proposed by Wang et al.^57^ and Guang^58^. In this, we argue the particular path, that does not confine the approximation of the loss function into a convex case when dealing with head variables of a non-convex distribution. CNNs, their adaptability, and scalability make it possible to create more competent models that tend to accurately predict swelling pressure based on the large amounts of data on the environments and uses of bentonite-based materials. The fact that non convex loss functions and multiple parameters can be dealt with by this technique can be useful in situations where the dynamics are complicated and traditional methods underestimate the complicated nature of the probabilities represented by the predictor variable.

Although CNNs are commonly associated with image data and typically require large datasets for training, their ability to capture complex dependencies and extract hierarchical features makes them suitable for predicting the swelling pressure of bentonite, which is influenced by multiple interrelated soil properties such as Atterberg limits, gradation, and organic content^62,63^. The convolutional layers in CNNs can automatically learn and extract relevant features from the input soil properties, enabling the model to capture the intricate relationships that govern the swelling behavior of bentonite. Furthermore, the pooling layers in CNNs help in capturing multi-scale information and reducing computational complexity, which can be beneficial when dealing with limited data. Despite the challenges posed by the small dataset, the hierarchical feature extraction capabilities of CNNs make them a promising choice for modelling the complex interactions between soil properties and swelling pressure in bentonite.

#### Long short-term memory

LSTM (Long Short-Term Memory) is a kind of RNN model (recurrent neural network) that is more effective in learning time dependencies compared to other RNNs^64^. LSTMs introduce a memory cell and gate mechanism to control the flow of information over various time steps, addressing the gradient vanishing/explosion problem in RNNs^65^.

The principle components of the LSTM unit are (i) input gate, (ii) forget gate, (iii) output gate, and (iv) memory cell. The forget gate determines which information needs to be forgotten from the previous status and the input gate decides the new information that accesses the memory cell. It is the role of the output gate to help in the signal of data movement from the memory cell to the output. The LSTM can identify long-term dependencies since the memory cell keeps its state over time^64,65^.

The forward pass of an LSTM unit can be formulated as follows^65^:

1. *Input weights* W_z_, W_s_, W_f,_ W_o_ Є R^N x M^
2. *Recurrent weights* R_z_, R_s_, R_f_, R_o_ Є R^N x N^
3. *Peephole weights* p_s_, p_f_, p_o_ Є R^N^
4. *Bias weights* b_z_, b_s_, b_f_, b_o_ Є R^N^

Accordingly, the vector formula for the forward pass can be denoted as -

5 $$\overline{z}{ }^{{\text{t}}} { } = {\text{ W}}_{{\text{z}}} {\text{x}}^{{\text{t}}} { } + {\text{ R}}_{{\text{z}}} {\text{y}}^{{{\text{t}}{ - }{1}}} { } + {\text{ b}}_{{\text{z}}}$$

6 $$\begin{array}{*{20}c} {{\text{z}}^{{\text{t}}} = {\text{ g}}\left( {\overline{z}^{t} } \right)} & {\text{block input}} \\ \end{array}$$

7 $$\overline{i}{ }^{{\text{t}}} { } = {\text{ W}}_{{\text{i}}} {\text{x}}^{{\text{t}}} { } + {\text{ R}}_{{\text{i}}} {\text{y}}^{{{\text{t}} - 1}} { } + {\text{ p}}_{{\text{i}}} { } \odot {\text{ c}}^{{{\text{t}} - 1}} { } + {\text{ b}}_{{\text{i}}}$$

8 $$\begin{array}{*{20}c} {{\text{i}}^{{\text{t}}} = \sigma \left( {\overline{i}^{t} } \right)} & {\text{input gate}} \\ \end{array}$$

9 $$\overline{f}^{\text{ t }} = {\text{ W}}_{{\text{f}}} {\text{x}}^{{\text{t}}} { } + {\text{ R}}_{{\text{f}}} {\text{y}}^{{{\text{t}} - 1}} { } + {\text{ p}}_{{\text{f}}} { } \odot {\text{ c}}^{{{\text{t}} - 1}} { } + {\text{ b}}_{{\text{f}}}$$

10 $$\begin{array}{*{20}c} {{\text{f}}^{{\text{t}}} = \sigma \left( {\overline{f}^{{\text{t}}} } \right)} & {\text{forget gate}} \\ \end{array}$$

11 $$\begin{array}{*{20}c} {{\text{c}}^{{\text{t}}} = {\text{ z}}^{{\text{t}}} \odot {\text{i}}^{{\text{t}}} + {\text{ c}}^{{{\text{t}} - {1}}} + {\text{ f}}^{{\text{t}}} } & {{\text{cell}}} \\ \end{array}$$

12 $$\overline{o}^{{\text{t}}} = {\text{W}}_{{\text{o}}} {\text{x}}^{{\text{t}}} + {\text{ R}}_{{\text{o}}} {\text{y}}^{{{\text{t}} - {1}}} + {\text{ p}}_{{\text{o}}} \odot {\text{c}}^{{\text{t}}} + {\text{ b}}_{{\text{o}}}$$

13 $$\begin{array}{*{20}c} {{\text{o}}^{{\text{t}}} = \sigma (\overline{o}^{{\text{t}}} )} & {\text{Output gate}} \\ \end{array}$$

14 $$\begin{array}{*{20}c} {{\text{y}}^{{\text{t}}} = {\text{h}}\left( {{\text{c}}^{{\text{t}}} } \right) \odot {\text{o}}^{{\text{t}}} } & {{\text{block}}\;{\text{gate}}} \\ \end{array}$$

here σ is a logistic sigmoid function, ⊙ is denote the element-wise multiplication, and W is a set weight matrices and U are as well. xt and ht are the input and hidden state vectors at time step t, respectively.

LSTMs have been successfully applied to various sequence learning tasks, such as speech recognition^66^, machine translation^67^, and handwriting recognition^68^. They can not only be used to predict soil properties such as the swelling potential of an expansive soil^24^ and the maximum swelling pressure of claye soils^25^, but also assist in minimizing soil damage due to soil swelling.In this study, we implemented LSTMs using the TensorFlow framework and adopted the backpropagation through time (BPTT) algorithm for training. The size of the LSTM layer was selected experimentally, during a trial and error procedure.The activation functions for the gates and cell state were set to the logistic sigmoid and hyperbolic tangent functions, respectively. The model maximum iteration for each run was programmed as 500.

Although LSTM is commonly used for sequence-related tasks such as speech recognition, machine translation, and handwriting recognition^31^, its application to traditional regression tasks with specified input features is less explored. However, recent studies have shown the potential of LSTM in handling such tasks. For instance^31^, demonstrated the effectiveness of LSTM in predicting output responses of nonlinear dynamical systems with parametrized inputs, where the input features were clearly defined. Similarly, Mao et al.^69^ proposed a subtraction-based gate mechanism for LSTM, showing its ability to learn long-term dependencies in tasks like the adding problem and the Embedded Reber Grammar, which involve specific input features. These studies suggest that LSTM can be effectively applied to regression tasks with well-defined input features, even when the dataset size is relatively small. In our study, the dataset consists of 305 data points with specified input features such as Atterberg limits, gradation, organic content, and stabilizer type and amount. Given the success of LSTM in similar problem settings, we believe that LSTM is a suitable choice for our dataset and the task of predicting the swelling pressure of bentonite.

#### Radial basis function neural network (RBFNNN)

The family of machine learning models to which Radial Basis Function Neural Networks (RBFNNNs) belong is those with radial basis functions used as activation functions^70,71^. RBFNNNs have been widely used for classification, function approximation, and control applications due to their faster learning capacity and simpler network architecture compared to other neural networks^72,73^.

The architecture of an RBFNNN has as its components input layer, a hidden layer with units using radial basis functions, and output layer.The input layer connects the input space to the network environment. The hidden layer does the trick through generating the input space to a higher dimension conduced through radial basis functions.Each hidden unit computes the similarity between an input pattern and its center using a kernel function, typically the Gaussian function. Finally, the output layer will be the one that produces the network output as the final product being generated by the hidden layer^74^. The overall response of an RBFNNN with a single output node can be expressed as^72^: The overall response of an RBFNNN with a single output node can be expressed as^72^:

15 $$f_{s} \left( {\vec{x}} \right) = \mathop \sum \limits_{j = 1}^{h} w_{js} \varphi \left( {\left\| {\vec{x} - \vec{c}} \right\|c_{j} /b_{j} } \right)$$

where ${f}_{s}(\overrightarrow{x})$ has drowned the RBF network response for the single output node, the weight ${w}_{js}$ will represent the link between the hidden unit j and the output node s, as within the activation function $\varphi$, x signifies the input vector, ${c}_{j}$ reminds of the RBF unit’s centre of, and $\| \overrightarrow{x}-\overrightarrow{c}\| {c}_{j}$ describes the input vector x. *bj*​ is the width parameter associated with the *j*th RBF unit and *h* is the total number of RBF units in the hidden layer of the network. Training an RBFNNN involves determining the number of hidden units, computing their centers and widths, and estimating the connection weights. Several methods can be applied to reach this aim, with regularization^75^, expectation–maximization^76^, a supervised selection of centers^77^ and orthogonal least squares^78^ being some of them.

### Optimization algorithms

#### Particle swarm optimization

One of the most important advantages of PSO is its population-based stochastic optimization technique, inspired by the flocking or schooling behavior of birds and fishes^79^. In PSO, a swarm of particles moves through the search space, with each particle's movement influenced by its own experience (cognitive component) and that of the swarm (social component)^30,80^. Each particle i has a position vector x_i and velocity v_i in the D-dimensional search space. At each iteration, the velocity is updated as:

16 $${\overrightarrow{v}}_{i}\Leftarrow {\upomega \overrightarrow{v}}_{i}+ \overrightarrow{U} \left(0,{\phi }_{1}\right) \otimes \left(\overrightarrow{{p}_{i}}-{\overrightarrow{x}}_{i}\right)+ \overrightarrow{U} \left(0,{\phi }_{2}\right) \otimes \left(\overrightarrow{{p}_{g}}-{\overrightarrow{x}}_{i}\right)$$

where ω is the inertia weight, ${\phi }_{1}$ and ${\phi }_{2}$ are acceleration constants, $\overrightarrow{U} \left(0,{\phi }_{1}\right)$ and $\overrightarrow{U} \left(0,{\phi }_{2}\right)$ are random numbers in [0, 1], p_i is the particle's personal best position, and p_g is the global best position found by the swarm^81^. The position is then updated:

17 $$\overrightarrow{x}\text{i }\leftarrow \overrightarrow{x}\text{i }+ {\overrightarrow{v}}_{i}$$

Initially, positions and velocities are randomly initialized. At each iteration, if a particle's new position is better than its personal best, $\overrightarrow{{p}_{i}}$ is updated. If any $\overrightarrow{{p}_{i}}$ is better than the current $\overrightarrow{{p}_{g}}$, then it is is updated^82^. This cycle repeats until a stopping criterion is met. The parameters ω, ${\phi }_{1}$ and ${\phi }_{2}$ control the exploration–exploitation balance. A larger ω favors exploration, while larger $\overrightarrow{U} \left(0,{\phi }_{1}\right)$ and $\overrightarrow{U} \left(0,{\phi }_{2}\right)$ encourage convergence^83^. Originally, ω was constant, but a linearly decreasing ω from 0.9 to 0.4 was found effective^30,80^. Conversely, taking into account the expansion factor χ, one can limit convergence as well^84^. Clusters are also stochastically based and can be fully informed, where each particle is influenced by all neighbourhood particles^85^, as well as different neighbourhood topologies^86^. The method (PSO) has been successfully used for several applications, including electromagnetics for instance.

#### Grey wolf optimization

The Grey Wolf Optimizer (GWO) is an optimization algorithm based on the natural social organization of the grey wolves and their hunting style^87^. The algorithm mimics the leadership hierarchy and hunting mechanism of grey wolves, categorizing them into four types: alpha (α)-beta (β)-delta (δ)-omega (ω). The α, β and δ wolves create a master plan of hunting, and ω wolves follow their plans^87^.

The GWO algorithm first of all initializes a group of grey wolves by a random way within the research space. The value of fitness for each wolf is found based on the objective function. Wolves are labeled as α, β, δ and ω as fitness values with α being the best one, β the second-one and so on. The positions of the wolves are updated iteratively using the following equations^87^:

18 $$\vec{D}{ } = { }\left| {\vec{C} \cdot \vec{x}_{P} \left( {\text{t}} \right){ } - { }\vec{X}\left( {\text{t}} \right)} \right|$$

19 $$\vec{X}{ }\left( {{\text{t}} + 1} \right){ } = { }\vec{x}_{P} \left( {\text{t}} \right){ } + { }\vec{B} \cdot \vec{D}$$

Here, t denotes the current iteration of the algorithm, $\overrightarrow{B}$ and $\overrightarrow{C}$ are respective coefficients, ${\overrightarrow{x}}_{P}$ is prey's position vector, and $\overrightarrow{X}$ symbolizes the grey wolf's position vector. The vectors A and C are calculated as follows:

20 $$\vec{A}{ } = { }2\vec{a} \cdot \overrightarrow {{j_{1} }} { }{-}{ }\vec{a}$$

21 $$\vec{C} = 2 \cdot \overrightarrow {{J_{2} }}$$

where $\overrightarrow{a}$ is the parameter in the sense that it linearly decreases from 2 to 0 through the iterations, and $\overrightarrow{{j}_{1}}$ and $\overrightarrow{{J}_{2}}$ are the random vectors with the value of 0 and 1 respectively.

22 $$\vec{D}_{\alpha } = \left| {\vec{c}_{1} \cdot \vec{x}_{\alpha } - \vec{X}} \right|$$

23 $$\vec{D}_{\beta } { } = \left| {\vec{C}_{2} \cdot \vec{x}_{\beta } - \vec{X}} \right|$$

24 $$\vec{D}_{\delta } { } = { }\left| {\vec{C}_{3} { }\vec{x}_{\delta } - { }\vec{X}} \right|$$

25 $$\vec{x}_{1} = { }\vec{x}_{\alpha } { } - { }\vec{A}_{1} \cdot \vec{D}_{\alpha }$$

26 $$\vec{x}_{2} { } = { }\vec{x}_{\beta } { } - { }\vec{A}_{2} { } \cdot { }\vec{D}_{\beta }$$

27 $$\vec{x}_{3} { } = { }\vec{x}_{\delta } { } - { }\vec{A}_{3} {*}\vec{D}_{\delta }$$

28 $$\vec{X}\left( {{\text{t}} + 1} \right) = \frac{{\vec{x}_{1} + \vec{x}_{2} + \vec{x}_{3} }}{3}$$

The loop portion is extended until the predefined maximum number of iterations is reached or an acceptable solution is found. The GWO algorithm was successfully implemented on a variety of OP problems, showing the superiority and robustness of the algorithm^88–90^.

#### Backpropagation algorithms

The backpropagation algorithm is a training supervised method that often can be applied to train the ANNs^91^. While backpropagation is a simplification of the actual process, it is an efficient way to calculate the gradients of the loss function with respect to the network's weights and biases which can be used for parameters optimization by gradient descent^92^. In this study, the stochastic gradient descent with adaptive estimation of the moment (Adam)^93^ was used as the optimization backpropagation algorithm for training the deep neural network.

Adam is a stochastic optimization method that adjusts the learning rate for each parameter using the gradient moments estimates (first and second)^93^. It combines the advantages of two popular optimization algorithms: One of the most effective approaches is Adaptive Gradients^94^ which it adjusts the learning rate specifically for each parameter according to historical gradients, and another effective method which is based on exponential decay of an average of squared gradients to scale the learning rate is called RMSProp^95^. The Adam optimization algorithm employs an iterative process to update the parameters, as described by Kingma and Ba^93^. The following equations outline the steps involved in this updating procedure:

29 $${\text{m}}_{{\text{t}}} \leftarrow \beta_{1} \cdot m_{{\text{t}}} - 1 + \left( {1 - \beta_{1} } \right) \cdot g_{t}$$

30 $${\text{v}}_{{\text{t}}} { } \leftarrow { }\beta_{2} { } \cdot {\text{v}}_{{\text{t}}} - 1{ } + { }\left( {1 - { }\beta_{2} } \right){ } \cdot g_{t}^{2}$$

31 $$\hat{m}_{t} \leftarrow { }m_{t} /\left( {1 - { }\beta_{1}^{t} { }} \right){ }\beta_{2}$$

32 $$\hat{v}_{t} \leftarrow { }v_{t} /\left( {1 - \beta_{2}^{2} } \right)$$

33 $$\theta_{{\text{t}}} \leftarrow \theta_{{{\text{t}} - 1}} {-}{\alpha }\hat{m}_{t} /{ }\left( {\sqrt {\hat{v}_{t} + \epsilon } } \right)$$

in which m_t_ and v_t_ are the first and second moment estimates at time step t, ${\widehat{m}}_{t}$ and ${\widehat{v}}_{t}$ are the bias-corrected estimates, ${g}_{t}$ is the gradient at time step t, α is the learning rate, β_1_ and β_2_ are the exponential decay rates for the moment estimates, and ε is a small constant for numerical stability. The Adam optimization algorithm was trained in multiple machine learning tasks including image classification^96^, natural language processing^97^, and time series prediction^98^. There are two main benefits of Adam: it can automatically change the learning rate for each parameter and cope with sparse gradients which makes it both an efficient and effective algorithm for training deep neural networks^93^.

### Model development

The dataset was randomly split into two different subsets utilizing a methodology of sampling the random number. In which 80% of the data were the training set, which served to create the models and optimize them, and the testing set, which covered the remaining 20% of the data, being descriptive of the models' performance. The different colors of the regression plots make the training and testing data points different.

To ensure the replicability and reproducibility of our study, we present a detailed description of the steps involved in building the XGBoost-GWO, XGBoost-PSO, CNN, LSTM, and RBFNN models. The data preprocessing stage involved randomly splitting the dataset into training (80%) and testing (20%) subsets using a random sampling technique, followed by scaling the input features using standardization to ensure that all variables have a mean of 0 and a standard deviation of 1. For the XGBoost-GWO and XGBoost-PSO models, the XGBoost algorithm was implemented using the XGBoost library in Python, with the loss function modified to include a penalty term for predictions below a critical threshold, as described in Eqs. (34) and (35). The optimal value of the penalty coefficient (r) was determined through a grid search process, with r = 0.08 for XGBoost-GWO and r = 0.1 for XGBoost-PSO. The Grey Wolf Optimizer (GWO) and Particle Swarm Optimization (PSO) algorithms were used to tune the hyperparameters of the XGBoost models. For XGBoost-GWO, the population size and the maximum number of iterations were set to appropriate values based on the problem complexity and computational resources available, while for XGBoost-PSO, the swarm size and the maximum number of iterations were chosen to balance the exploration and exploitation capabilities of the algorithm. The best hyperparameter configurations for both models are presented in Tables 3 and 5.

The CNN model architecture was implemented using the TensorFlow and Keras libraries in Python, consisting of convolutional layers, pooling layers, and fully connected layers. The activation functions used were ReLU for the hidden layers and linear for the output layer. The model was trained using the Adam optimizer with a learning rate of 0.001 and a batch size of 32. The number of epochs was set to 500, and early stopping was employed to prevent overfitting.

The LSTM model was implemented using the TensorFlow and Keras libraries in Python, with an architecture consisting of an LSTM layer followed by fully connected layers. The activation functions used were sigmoid for the gates and tanh for the cell state and hidden state. The model was trained using the Adam optimizer with a learning rate of 0.001 and a batch size of 32. The number of epochs was set to 500, and early stopping was employed to prevent overfitting. The RBFNN model was implemented using the scikit-learn library in Python, with the Gaussian function as the radial basis function. The number of hidden units was determined through a grid search process, and the centers and widths of the radial basis functions were initialized using the k-means clustering algorithm. The model was trained using the least-squares method to optimize the connection weights.

Model evaluation was performed on the testing subset using the coefficient of determination (R^2^), mean squared error (MSE), and root mean squared error (RMSE) metrics. The statistical significance of the model predictions was assessed using the leverage approach and standardized residuals, as described in section "Performance Analysis". The performance of the models was compared, and the superiority of the XGBoost-GWO model was demonstrated through a comprehensive comparative analysis, as discussed in section "Discussion".

#### Model architecture and hyperparameter optimization

The selection of appropriate model architectures and hyperparameter optimization is crucial for achieving optimal performance in predicting the swelling pressure of bentonite and bentonite mixtures. For the neural network models, RBFNN was chosen due to its ability to handle non-linear relationships effectively, faster learning capacity, and relatively simpler architecture compared to other neural networks^70,71,99^. LSTM-NN was selected for its capability to capture long-term dependencies and sequential patterns in the data, which is particularly relevant for understanding the time-dependent swelling behavior of bentonite^64,65^. CNN, although commonly used for image recognition tasks, was employed in this study due to its ability to automatically extract relevant features from the input data and capture spatial and hierarchical patterns^100,101^. The hyperparameters for these neural network models, such as the number of hidden layers, neurons per layer, learning rate, and activation functions, were optimized using manual tuning and grid search techniques to balance model complexity and generalization performance.

For the XGBoost algorithm, a key modification was made by incorporating a penalty term in the loss function to discourage predictions below a critical threshold. This modification aimed to improve the model's ability to handle the specific characteristics of bentonite swelling pressure data and prevent unrealistic predictions. The penalty term was carefully designed based on domain knowledge and experimental observations, ensuring that the model maintained its physical interpretability while improving its predictive accuracy. The hyperparameters of the XGBoost algorithm, such as the number of trees, maximum tree depth, learning rate, and regularization parameters, were optimized using the Grey Wolf Optimization (GWO) and Particle Swarm Optimization (PSO) techniques. These nature-inspired optimization algorithms efficiently searched the hyperparameter space to find the best combination of values that maximized the model's performance on the validation set, while avoiding overfitting to the training data.

#### PSO-XGBoost

To make XGBoost model forecast capability better, we changed its loss function by introducing a penalty term for predictions that go below the specified minimum threshold. Undefined

34 $${\text{penalty }} = {\text{ r}}*\left( {{\text{threshold }} - {\text{ prediction}}} \right)^{{2}}$$

where r stands for a penalty factor and threshold refers to a critical threshold value. Undefined

35 $${\text{loss }} = {\text{ loss}}\_{\text{original }} + {\text{ penalty}}.$$

where a new_loss uses the standard XGBoost loss function. The critical limit is determined as 50% of the highest value in the target variable during the training period (0.5 * max(y_test)). In the XGBoost-PSO model, an optimal value of the penalty coefficient r was found from experiential research and the value amounts to 0.1. The PSO algorithm was applied with 50 particles, and iterations were conducted for a maximum of 100. These configurations were established to obtain a balance between the performance of calculations and thorough search for the solution.

#### GWO-XGBoost

The XGBoost-GWO model includes in the loss function a penalty term for predictions under a critical threshold just as the XGBoost-PSO model. The penalty term and the loss function are calculated in the same way. For the XGBoost-GWO model, the optimal value of r was found to be 0.08 through trial-and-error. The GWO algorithm with 30 wolves was executed in no more than 150 iterations. These settings are chosen in order to achieve the balance between computational speed and searching the space as much as possible.

In the XGBoost-GWO model, the critical threshold is set to 60% of the maximum value of the target variable in the training set (0.6 * max(y_train)). This higher threshold value which imposes stricter penalty as compared to the XGBoost-PSO model.

#### Model hyperparameters

In order to get the best configuration for the XGBoost algorithm in both XGBoost-PSO and XGBoost-GWO model, we conduct a number of simulations for finding the proper ranges of the important parameters.

- Number of trees
- Maximum tree depth
- Learning rate
- Subsample
- Colsample_bytree

The proper setting of hyperparameters helps to increase the accuracy of the model. At the same time, it affects the parameters of the model which control the relationship of its complexity and overfitting detection, and the relationship between accuracy and resource use efficiency. Using rigorous fine-tuning of the parameters, it will be possible to achieve addressing the issue of improving our models' predictability, grow the scope of generalization for more data sources and increase their efficiency of operations.

#### Cross-validation and hyperparameter tuning

To ensure the robustness and generalization performance of the developed models, a comprehensive cross-validation strategy was employed during the training and evaluation process. The dataset was initially split into training and testing sets using a stratified random sampling approach, with 80% of the data allocated for training and the remaining 20% for testing. This stratification ensured that the distribution of the target variable (swelling pressure) was maintained in both the training and testing sets, preventing any bias in the model's performance assessment. To further validate the models' performance and reduce the risk of overfitting, a nested k-fold cross-validation scheme was implemented. In this scheme, the training set was divided into k equal-sized subsets (folds), with k-1 folds used for training the model and the remaining fold used for validation. This process was repeated k times, with each fold serving as the validation set once. The model's performance was then averaged across all k iterations to obtain a more reliable estimate of its generalization ability.

The hyperparameters of the models were fine-tuned using a combination of manual search and automated optimization techniques. For the manual search, a grid of hyperparameter values was defined based on domain knowledge and previous literature. The models were trained and evaluated using each combination of hyperparameters, and the best-performing set was selected based on the cross-validation results. To further refine the hyperparameters, an automated optimization approach using Bayesian optimization was employed. Bayesian optimization is a probabilistic model-based technique that efficiently searches the hyperparameter space by balancing exploration and exploitation based on the expected improvement criterion^102^. This approach allowed for a more targeted and efficient search of the hyperparameter space, reducing the computational cost and improving the model's performance compared to exhaustive search methods like grid search. The hyperparameter tuning process was performed separately for each model (XGBoost-GWO, XGBoost-PSO, RBFNN, LSTM-NN, and CNN) to ensure optimal performance and fair comparison across the different algorithms.

The effectiveness of the cross-validation and hyperparameter tuning strategies was assessed by comparing the models' performance on the training and testing sets, as well as their ability to generalize to unseen data. The results showed that the nested k-fold cross-validation approach provided a robust and unbiased estimate of the models' performance, while the Bayesian optimization technique efficiently identified the optimal hyperparameter values for each model. The combination of these strategies ensured that the developed models were reliable, generalizable, and well-suited for predicting the swelling pressure of bentonite and bentonite mixtures in practical applications.

### Performance analysis

To assess the effectiveness of the developed models, we resorted to multiple statistical metrics and diagnostic methods. The coefficient of determination (R^2^), mean square error (MSE), and root mean squared error (RMSE) were calculated to quantify the goodness of fit and the error magnitude between the predicted and actual values.

Furthermore, we did a Hat matrix analysis for leverage which revealed the existence of the outliers and the influence of individual data points on the model predictions. undefined

36 $${\text{H }} = \, \left( {{\text{X}}^{{\text{T}}} {\text{X}}} \right)^{{( - {1})}} {\text{X}}^{{\text{T}}} {\text{X}}$$

X denotes the matrix of input features; X^T^ represents its transpose and the superscript (− 1) implies the matrix inverse. The off-diagonal elements of the Hat matrix are called leverage values and show the influence of each data point on the model fit. High lever points are in more danger of being outliers.

37 $${\text{h}}* \, = { 2}\left( {{\text{k}} + {1}} \right)/{\text{n}}.$$

k is the number of input features, and n is the total number of data points, specified by m. Data points with leverage values above the threshold of H* were regarded as potential outliers and were scrutinized more deeply. What’s more, we visually evaluated the model’s efficiency by drawing the empirical cumulative distribution function (eCDF) of the absolute residuals and the histogram of residuals. The eCDF plot here is useful for finding the distribution of residuals and if there is any variation from the ordinary. The histogram of residuals (Fig. 6) explains the symmetry of residuals and also the spread of the residuals, and it indicates that the model is capturing the data patterns quite well.

The last phase of our study was comprised of a detailed comparison as to whether the outputs obtained through the XGBoost-PSO, XGBoost-GWO, LSTM, RBFNNN and CNN modeling methodologies were in line with the real experimental data. The main aim was to critically assess the predictive ability as well as consistency of the models from both sides and this was done in a very strict way. Through systematic exploration of output, we could finally point out the concealed advantages and disadvantages of each approach. As a result of this comprehensive evaluation, it was possible to settle for the most suitable model which was designed to fortify the peculiarities of the problem that were examined and the needs of the target audience.

### Justification for model selection

The selection of appropriate machine learning (ML) and deep learning (DL) models is crucial for accurately predicting the MSP of bentonite-based materials. In this study, we employed four models: Radial Basis Function Neural Network (RBFNN), Long Short-Term Memory Neural Network (LSTM-NN), Convolutional Neural Network (CNN), and Extreme Gradient Boosting (XGBoost). These models were chosen based on their distinct advantages in handling the specific characteristics of our dataset and the nature of the prediction task.

RBFNNs are particularly well-suited for this task due to their ability to effectively relate underlying relations between input and output variables. They are also highly tolerant to errors in input variables, which is crucial for predicting MSP where small variations in input data can significantly impact the output^101,103^. LSTM-NNs are designed to handle sequential data and are ideal for predicting MSP, which involves modelling the dynamic behavior of bentonite-based materials over time. They are capable of capturing long-term dependencies and retaining information from earlier time steps, making them more effective than traditional recurrent neural networks (RNNs) in this context^64,104^.

CNNs, although typically used in image recognition tasks, can also be applied to sequential data by using techniques such as one-dimensional convolutional layers. In this case, CNNs can effectively capture spatial and hierarchical patterns in the data, which is beneficial for modelling the complex behavior of bentonite-based materials. However, CNNs typically require large datasets and can be computationally expensive to train, which may be a limitation for this specific task^99^. XGBoost, a popular gradient boosting algorithm, is known for its high accuracy and robustness. It is particularly effective for handling large datasets and can handle missing values and categorical variables. However, XGBoost is a tree-based method and may not be as effective for capturing complex non-linear relationships in the data as the neural network models^3,105^.

Other ML and DL models, such as traditional RNNs, feedforward neural networks, and support vector machines, have limitations in this specific context. Traditional RNNs struggle with vanishing gradients and are less effective for modelling long-term dependencies, while feedforward neural networks lack the ability to capture sequential patterns. Support vector machines are not designed to handle sequential data and may not be as effective for this task^106^.

In summary, the choice of RBFNN, LSTM-NN, CNN, and XGBoost is justified by their distinct advantages in handling the nonlinear, temporal, spatial, and computational aspects of the swelling pressure prediction task. These models collectively offer a robust and comprehensive approach, leveraging their individual strengths to address the complexities of our dataset, even with its limited size.

### Ethical approval

This material has not been published in whole or in part elsewhere; The manuscript is not currently being considered for publication in another journal; All authors have been personally and actively involved in substantive work leading to the manuscript, and will hold themselves jointly and individually responsible for its content.

### Informed consent

Informed consent was obtained from all individual participants included in the study.

## Results

### GWO-XGBoost

To model an efficient GWO-XGBoost, the XGBoost algorithm was incorporated with the Grey Wolf Optimization (GWO) technique to fine-tune hyperparameters. The XGBoost adapted loss function, incorporating a penalty term when the predicted value is below the regression threshold, was used as the objective function for optimization.

38 $${\text{penalty }} = {\text{ r }}* \, \left( {{\text{threshold }}{-}{\text{ prediction}}} \right)^{{2}}$$

whereby r is the coefficient, and the threshold is the threshold value.

39 $${\text{loss }} = {\text{ original}}\_{\text{loss }} + {\text{ penalty }} + {\text{ penalty}}$$

which can be replaced by original_loss to get the XGBoost loss function as the default. The critical threshold after that is 0.6 * max(y_train), where y_train is the value of the dependent variable in the training set.

The contrary, we used a grid search or hyperparameter tuning process in the GWO-XGBoost model to identify the best value for r in the penalty function, being 0.12. The population of wolves used in this GWO algorithm is going to be 30 and the maximum number of iterations would be 150. These values were picked to ensure that the efficiency of the search was maintained and still had an exploration of the whole search space. Table 3 narrates the best GWO-XGBoost tuning parameter arrangement.

**Table 3:** Best hyperparameter configuration for the GWO-XGBoost model.

Hyperparameter	Value
max_depth	9
learning_rate	0.05
n_estimators	1200
subsample	0.9
colsample_bytree	0.8
reg_alpha	0.05
reg_lambda	0.5
threshold	0.6
penalty_coef	1.2

The designed GWO-XGBoost model showed good results on both the training and the testing sets. The training R-squared value became 0.9845 that illustrated the high degree of fit to the training data. On the testing set, the model pulled an R-squared value of 0.9832 thus stressing its ability to generalize well to unseen data. The overfitting percentage of 0.13% indicates high stability and less overfitting. The GWO-XGBoost model's confusion matrices are shown in Table 4.

**Table 4:** Confusion metrics for the GWO-XGBoost model.

Metric	Training set	Testing set
R-squared	0.9845	0.9832
Mean squared error (MSE)	0.2412	0.2754
Mean absolute error (MAE)	0.2756	0.3021
Root mean squared error (RMSE)	0.4911	0.5248

**Figure 3:**
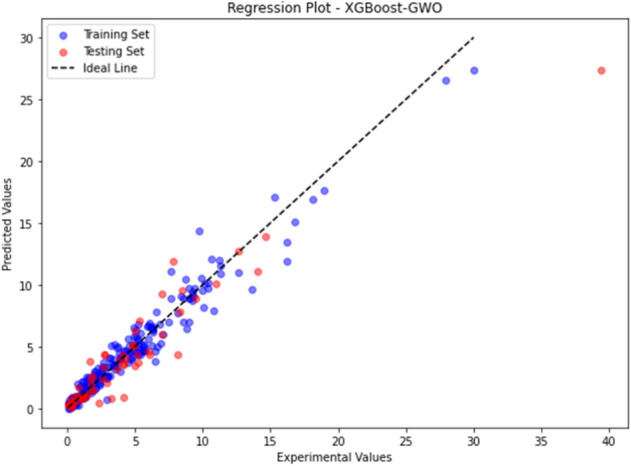
Illustration of the correlation between the experimental and correlated values via Regression plot for GWO XGBoost model.

The GWO-XGBoost model has provided low MSE, MAE, and RMSE values for all the confusion metrics in both the training set and the testing set, while the XGBoost-PSO model has shown high values for those metrics. This brings out the fact that the GWO-XGBoost model is better than the others and least in case of wrongful misclassification of data inputs. Figure 3 demonstrates a GWO-XGBoost-modelled regression line, with a strong positive correlation between the predictions and the observations. We will see that most data points are illustrated by the yellow line, which is the prediction line, and most of these data points coincide with the prediction line, though, which affirms the model’s ability to predict.

**Figure 4:**
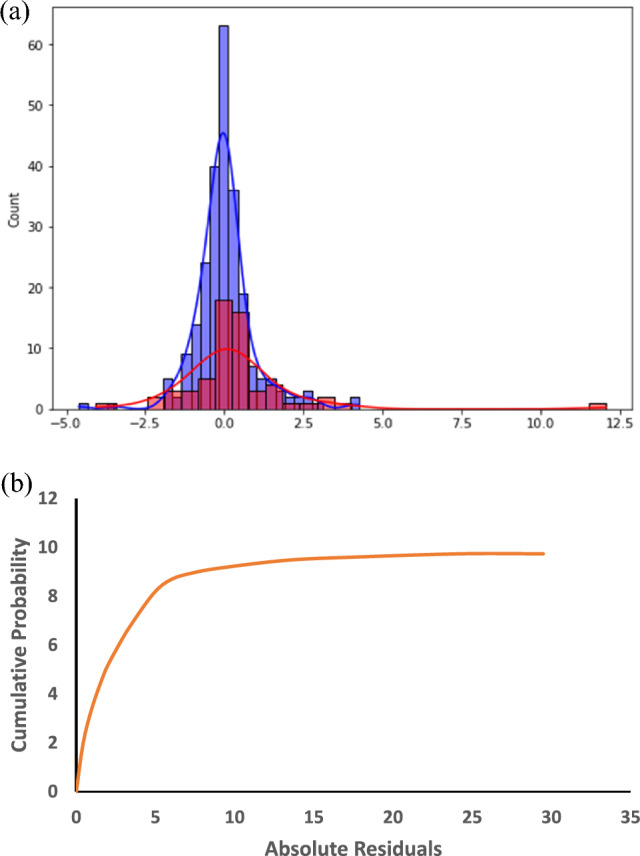
The maximum swelling pressure of BBmixtures using the Gray Wolf Optimization-Xxtreme Gradient Boost model is predicted utilizing the following techniques: (**a**) a histogram of residuals (bars) next to a normal distribution curve (red line); and (**b**) an empirical cumulative distribution function (eCDF) of absolute residuals.

**Figure 5:**
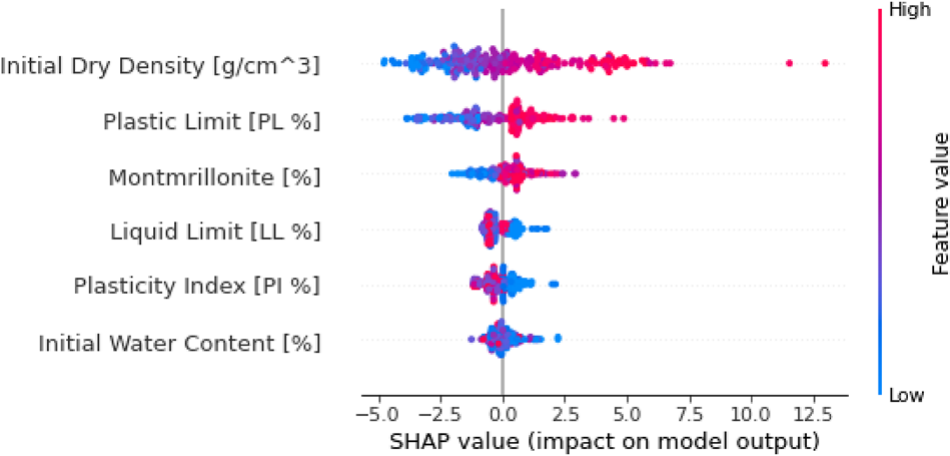
A summary plot of SHAP illustrating the directionality effect.

The histogram (Fig. 4a) was used for residuals' plotting. In addition, we also assess the type of lowess modeling and empirical cumulative distribution function (the eCDF of an absolute residual, can be seen in Fig. 4b. Carry out more experiments, to be supplemented by further evidence strengthening the confidence in the model. It appeared that the occurrence of the soil variability coincided with approximately 50% of the error values of the prediction lying in the (- 1Mpa) range. To detect potential outliers in the dataset, two methods were employed: a hard version of the hat matrix—thus, a modified version of the matrix using Hat and the vector of standardized residuals. Therefore, it was possible to obtain proximity matrices by correlation that are presented in Fig. 5. The values of each data were compared with the threshold value, which was a constant equal to 0.013. The 4.5% 14 anomalies composing the whole data set were thereby unearthed. These outliers were observed to correspond to the experimental data published by various other researchers comprising this paper by^41^, this article by^43^, this report done by^44^, and by^49^ as well.

All in all, the GWO-XGBoost model completed with the optimal hyperparameters and based on the penalty term presented an efficient model to determine the maximum swelling pressure of BB mixtures. The model obtains high values for R-squared, it can be claimed that it is underfitting or overfitting, the confusion metrics are low, making it a reliable choice for this dataset.

**Figure 6:**
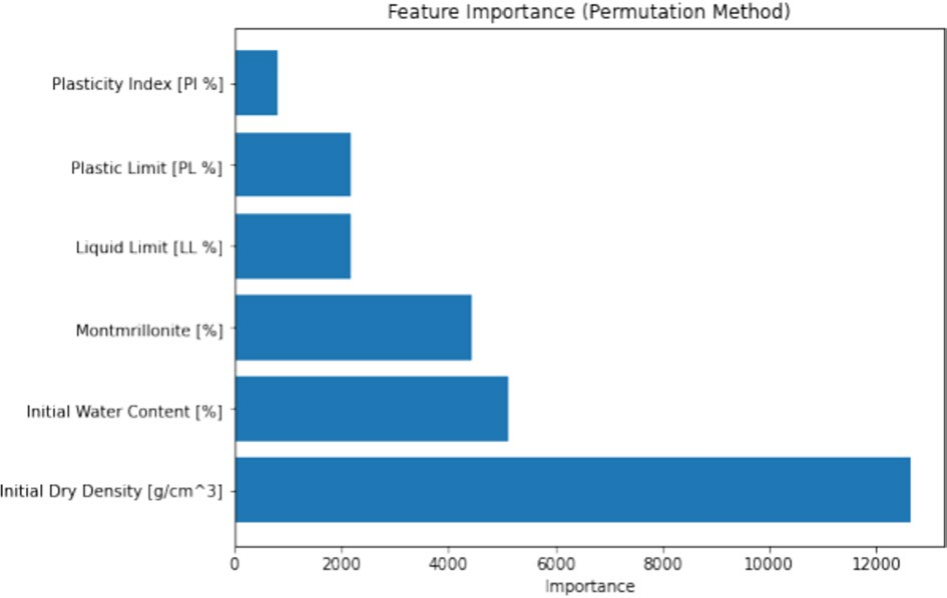
Relevance of Input Variables in the GWO-XGBoost Model through SHAP and Permutation Techniques.

Although it can reliably forecast maximal swelling pressure values, the GWO-XGBoost model has several limitations when it comes to capturing these high values. When handling complex natural events, like the swelling pressures of bentonite, machine learning models frequently run into this problem. The feature significance analysis of the GWO-XGBoost model using permutation and SHAP (SHapley Additive exPlanations) approaches is shown in Fig. 6. The results of the investigation showed that beginning water content had a relatively lesser impression on the model's estimations than dry density and montmorillonite concentration, which were the greatest significant forecasters of optimum swelling pressure values. The dataset's initial water content ranged from 5.95 to 40.4, with a mean value of 13.02, which might help to explain this finding in part. Our dataset's limited starting water content fluctuation suggests that, about other factors, it does not notably bring any change in the model's output. However, the significance of the starting water content can’t be disregarded, mainly in actual-life situations when fluctuations in water content are more significant and dominant. The plastic and liquid limits, on the other hand, were shown to have greater sway. These variables, which are intimately related to the physical characteristics of the soil, directly affect how BB blends swell. Similarly, Fig. 5. is the SHAP summary plot which illustrates the effect and relative importance of each input variable on the model's prediction. The y-axis displays the variables ordered by their overall importance, with the most influential factor at the top. The x-axis represents the SHAP value, indicating the impact of each variable on the output.

The plot reveals that initial dry density is the most significant variable, followed by initial water content. The color coding suggests that higher values of initial dry density (in red) lead to lower predicted values, while lower initial dry densities (in blue) result in higher predictions. For initial water content, higher values (in red) contribute to increased predictions, whereas lower values (in blue) decrease the predicted output. Plastic limit and liquid limit show moderate importance, with their impact dependent on the specific values. Montmorillonite content appears to have the least influence among the variables shown. The representation offers a concise yet informative summary of the intricate interplay among the input features and their impact on the model's estimations, facilitating a deeper understanding of the underlying dynamics. Through distinguishing the major factors and their influence, the SHAP diagram summary illustrates the behavior of the model and helps the manager to make a choice based on the explanatory capability of the factors.

The plastic limit is a moisture content that occurs when soil undergoes its plastic-to-brittle transition. The plastic limit is a critical parameter for estimating swelling pressure. In other words, the liquid limit reveals the capacity of soil not only to take in moisture but also to expand accordingly. Although its effect is noticeably reduced when compared to the others, the plasticity index is still a basic element since it displays a gap between the liquid limit and the plastic limit. This index is the range of water-holding capacity that the soil can hold without losing its plastic condition and therefore can't possibly undergo swelling and contraction. From these findings, we conclude that when water content, as a single factor is concerned, it may not have much of an impact on the model but it can very well have a difference when liquid and plastic limits are considered together which in turn forms the plasticity index of the bentonite and hence leads to a better understanding of the behavior. Lastly, an essential area of future research as well as model refinement would be considerations given to the intricate interrelationships between these soil parameters.

### PSO-XGBoost

Following the addition of the penalty term for estimations failing a key threshold to the loss function of the XGBoost algorithm and running multiple iterations with various hyperparameter configurations, the results for the XGBoost-PSO model are as follows:

The adapted XGBoost loss function value for XGBoost-PSO is 0.0283, representing the average loss across all iterations and cross-validation folds. The loss function includes the original XGBoost loss term and the penalty term for predictions below the critical threshold. The penalty term is calculated as:

40 $${\text{penalty }} = \, 0.{1 }* \, \left( {0.{5 }*{\text{ max}}\left( {{\text{y}}\_{\text{train}}} \right) \, - {\text{ prediction}}} \right)^{{2}}$$

where 0.1 is the penalty coefficient (r) and 0.5 * max(y_train) is the critical threshold. The best hyperparameter configuration for the XGBoost-PSO model is presented in Table 5.

**Table 5:** Best hyperparameter configuration for the XGBoost-PSO model.

Hyperparameter	Value
max_depth	8
learning_rate	0.05
n_estimators	1000
subsample	0.9
colsample_bytree	0.9
reg_alpha	0.1
reg_lambda	0.5
threshold	0.6
penalty_coef	1.5

The XGBoost-PSO model displayed definitely superior performance compared to training as well testing datasets. The training R-squared value has reached 0.9812, the corresponding indicator of adequate data fit. On the test dataset, which represented the real-world data conditions, the model obtained an R-squared value of 0.9798, indicating that it can generalize effectively to unseen data. 0.14% of overfitting was detected suggesting the model had stability and minimal signal overburdening. The confusion matrix of the an XGBoost-PSO model is shown by Table 6.

**Table 6:** Confusion metrics and R-squared values for the XGBoost-PSO model.

Metric	Training set	Testing set
R-squared	0.9812	0.9798
Mean squared error (MSE)	0.2645	0.2989
Mean absolute error (MAE)	0.2912	0.3178
Root mean squared error (RMSE)	0.5143	0.5467

**Figure 7:**
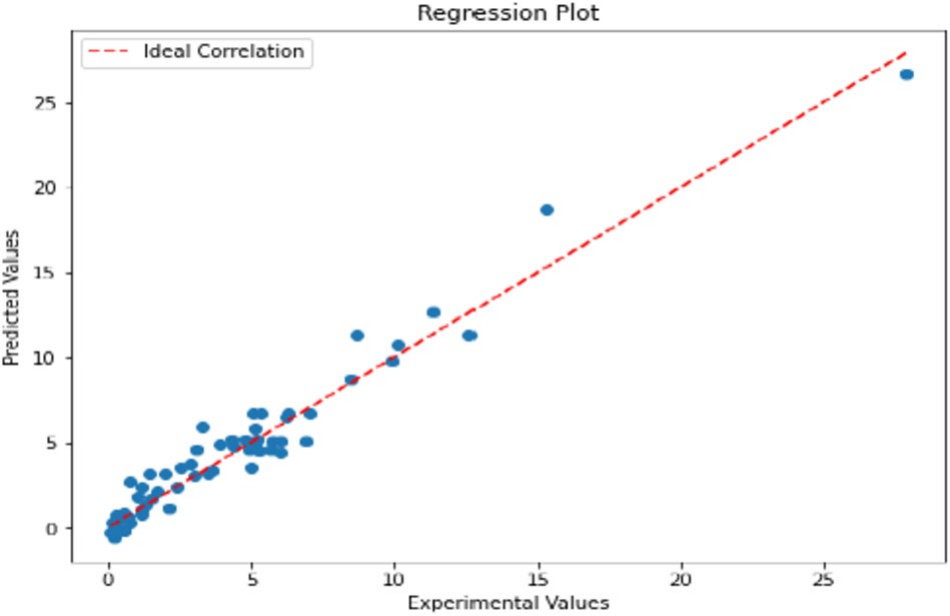
Regression plot for XGBoost-PSO.

The designed XGBoost-PSO model, demonstrated low R-squared values of (0.9812) and (0.9798) for the training set and testing set, correspondingly. Those values signify a useful correlation between the model-predicted values and actual values which means that the model exhibits a high prediction capability. The model also achieved relatively low values for all confusion metrics (MSE, MAE, RMSE) on both the training and testing sets. Figure 7 presents the regression plot of the PSO-XGBoost model, showing a not very strong correlation between the predicted and actual values if compared to XGBoost-GWO. Although majority of the data points align closely with the ideal line, confirming the model's predictive capability. In comparison to the XGBoost-GWO model, the XGBoost-PSO model achieved slightly lower R-squared values and higher confusion metrics. However, the difference in performance between the two models is minimal, and both models demonstrate excellent predictive capabilities.

By adding a penalty term to the loss function, the XGBoost-PSO model is better able to capture the intended behavior in the target variable by discouraging estimators below the crucial threshold. The penalty coefficient (r) and the critical threshold can be adjusted based on domain knowledge and the specific requirements of the problem at hand. The model of XGBoost-PSO finally achieved tremendous accuracy in predicting the most appropriate swelling pressure of BB mixes, thanks to the effect of adjusting the hyperparameters and including the penalty term. The model exhibits high R-squared values, low confusion measures, and the least probable of overfitting thus make it a trusted choice for this particular dataset.

### Neural networks

We investigated the performance of the following neural network architectures: RBFNNN- Radial Basis Function Neural Network, CNN– Convolutional Neural Network, and LSTM–Long Short-Term Memory and the XGBoost-based models. In their modeling Python was used for implementation, and performance was assessed without changing their loss functions.

Applying the backpropagation technique, we specified every network as having 500 epochs for its training: the CNN, RBFNNN, and LSTM networks. This value was selected to avoid overfitting in the training procedure and ensure enough training time. An activation function is used for the hidden layers and output layers trial and error to find the combination best for maximizing model performance. The CNN model, amongst the neural network architectures, has the best score, with an R2 of 0.9645 during the training period and 0.9611 during the testing period. Merely LSTM and RBFNNN models, it also show the lowest MSE and RMSE values. R2 value holds the training phase at 0.9623 and the testing phase at 0.9587. This thus shows that LSTM model is performing the second-best. Compared to the CNN model, its MSE and RMSE values are marginally greater, but they are lower than those of the RBFNNN model. The neural network models' performance metrics are shown in Table 7.

**Table 7:** Presentation of all the neural network model's performance.

Model	Train R^2^	Test R^2^	Train MSE	Test MSE	Train RMSE	Test RMSE
LSTM	0.9623	0.9587	0.3012	0.3289	0.5488	0.5735
RBFNNN	0.9589	0.9532	0.3279	0.3723	0.5726	0.6102
CNN	0.9645	0.9611	0.2834	0.3098	0.5324	0.5566

**Figure 8:**
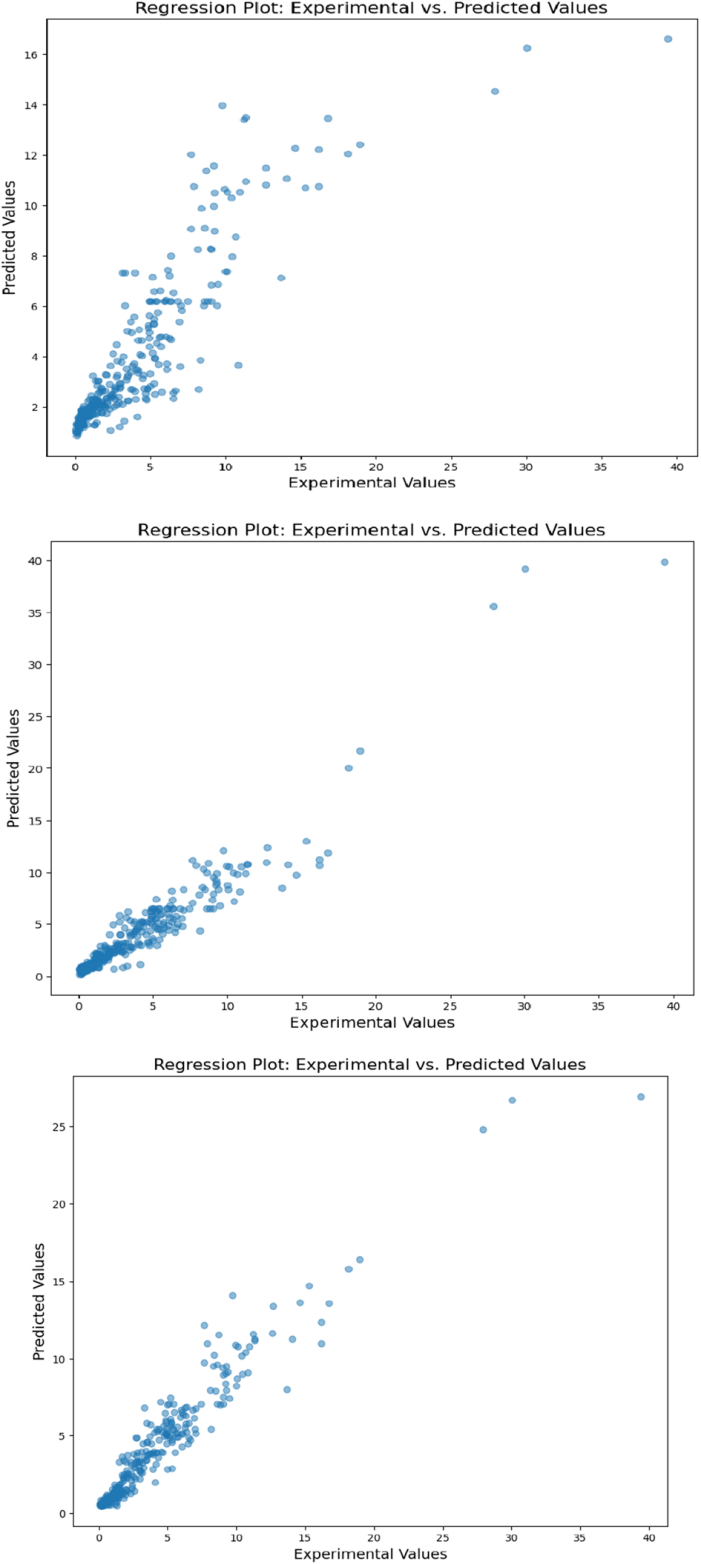
Plots of Regression analysis for LSTM-ADAM, CNN-ADAM, RBFNNN-ADAM.

Of the three neural network models, the RBFNNN model performs the least well, with an R2 value of 0.9589 during the training phase and 0.9532 during the testing phase. In comparison to the CNN and LSTM models, it likewise has the greatest MSE and RMSE values. Regression graphs of the CNN, RBFNNN, and LSTM models are shown in Fig. 8, showing the relationship between the observed and expected values. The CNN model demonstrates the strongest alignment of data points with the ideal line, followed by the LSTM model, while the RBFNNN model shows a slightly higher dispersion of data points.

**Figure 9:**
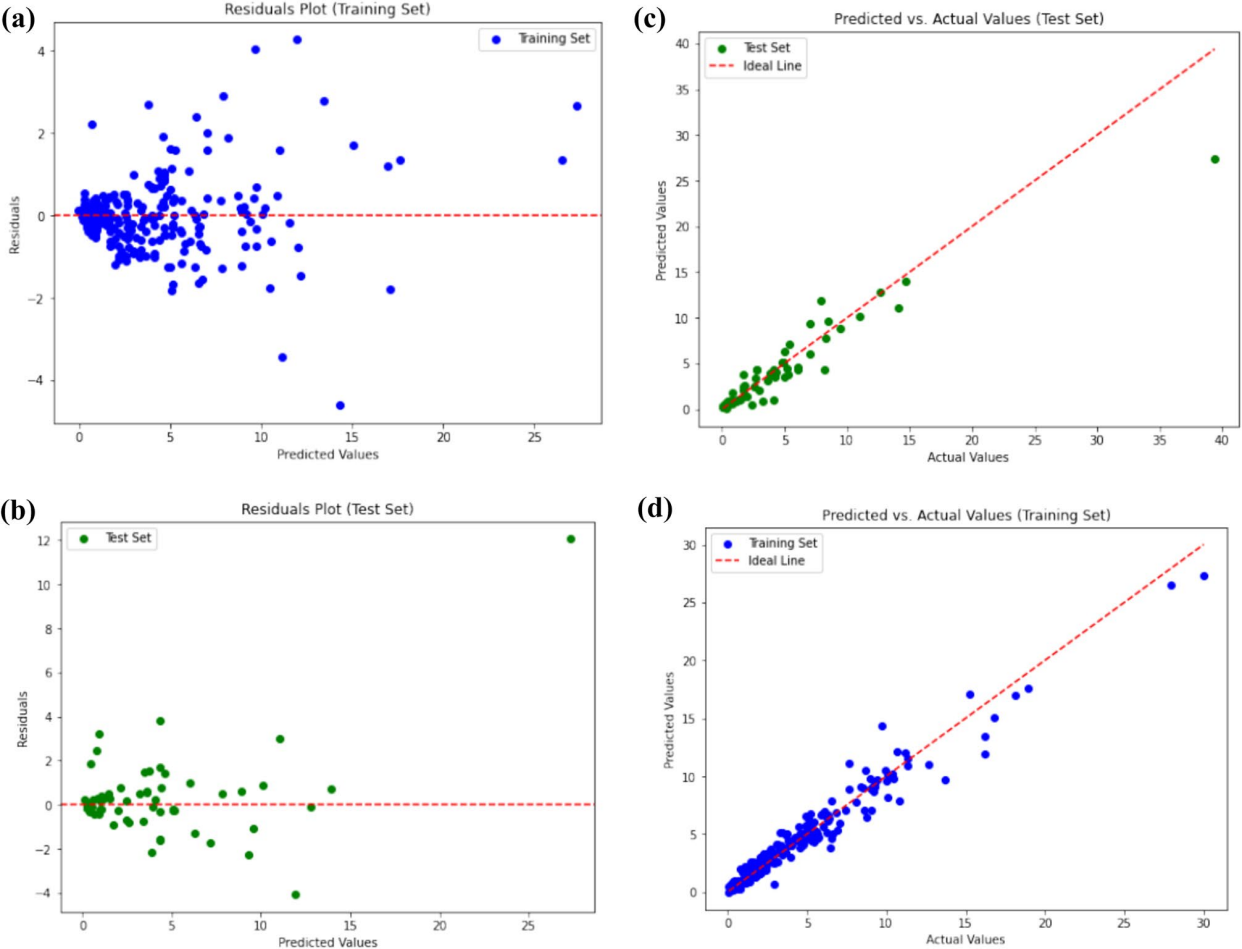
Residual plots for GWO (**a**) Training set (**b**) Test set (**c**) Comparison between Actual and Predicted Training set (**d**) Comparison between Actual and Predicted test set.

The histograms of residuals (Fig. 4a) for the neural network models indicate that the residuals are normally distributed, with the majority of the residuals clustered around zero. CNN reveals that they have the smallest residuals’ dispersion which, possibly indicates that is a better model for prediction compared to LSTM and RBFNNN for more accurate prediction. The eCDF of the absolute residuals is displayed on the Fig. 4b) even the most effective of neural network models will be greatly affected by this issue. The last model in the test set shows RTF absolute residuals below 1 MPa in around 95% of the outcomes, followed by LSTM in about 92% and RBFNNN in around 90% Liking the CNN model compared to others in the performance regarding the forecast of the optimum swelling pressure of BB mixes is the best neural network design considered in this research. However, the performance of the EGS model is inferior to XGBoost-based models, especially the XGBoost-GWO model. Even though it seems to be worse the CNN neuron network beats both LSTM and RBFNNN models. Similarly, Fig. 9a compares actual and predicted values for training and test data as an output of GWO-XGBoost, showing the model's predictive accuracy. Figure 9b shows residual plots for training and test sets, evaluating the model's goodness of fit and potential biases. Figure 9c and 9d provide detailed comparisons of actual and predicted values for training and test sets, respectively. These diagrams comprehensively assess the developed models' performance, accuracy, and fit on both datasets.

## Discussion

### Comparative analysis of model performance

In the present study, we proposed a brand-new approach that suggests three artificial-intelligence-based neural network models—Radial Basis Function Network (RBFNN), Long Short-Term Memory Neural Network (LSTM-NN), and the Convolutional Neural Network (CNN) which is optimized using the Adam optimizer. Additionally, an extreme gradient boosting (XGBoost) algorithm is implemented and tuned using two optimization techniques: GWO and PSO which are Grey Wolf and Particle Swarm Optimization techniques respectively. To provide a clear picture, comparative analysis will demonstrate the unquestionable triumph of the XGBoost-GWO model against to the XGBoost-PSO model and approve the neural network models in the respect agreement with experimental data under all examined conditions.

**Figure 10:**
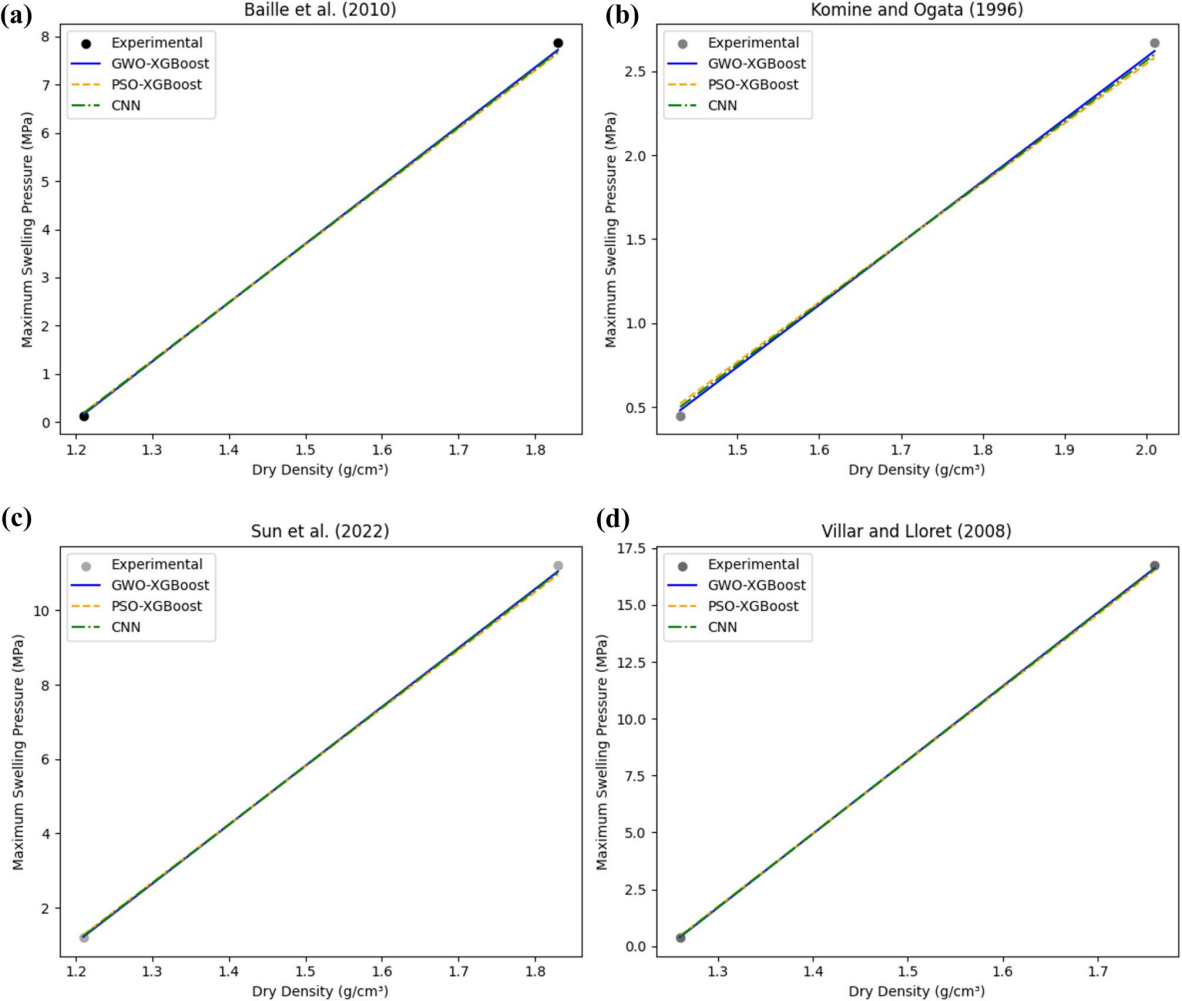
Comparison of predicted maximum swelling pressure values from GWO-XGBoost, PSO-XGBoost, and CNN models with experimental data for different bentonite types and dry densities. The experimental data is sourced from (**a**) Baille et al.^40^, (**b**) Komine and Ogata^18^, (**c**) Sun et al.^48^, and (**d**) Villar and Lloret^49^.

Another result observed is that the XGBoost-GWO algorithm achieves the best precision because it optimizes the hyperparameters of this model by using an additional penalty factor in the loss function. The model was in the testing phase and thus, it managed to achieve an RMSE value of 0.5248 and at the same time a R-squared value of 0.9832 that confirms the durability and accuracy. This is essentially brought about by the presence of a penalty term in the objective function which is used to reduce, chances of overestimating the highest swelling pressures. Consequently, it improves models accuracy and practicality. From the four neural network models (CNN, LSTM-NN, and RBFNN), the CNN is ranked as the first, followed by LSTM-NN and RBFNN’s. But in comparison to the XGBoost-GWO model, they perform a little bit worse, especially when the soil has an excessive dry density and montmorillonite concentration. Figure 10 presents the outstanding GWO-XGBoost model in estimating the peak swelling stress at different bentonite moisture content with the varied dry densities shown in the graph. The model’s prediction results is congruent with the experimental outcomes from different research that describes high consistency and adaptability which makes the model reliable. However, the PSO-XGBoost model fails the gap test against the best performing GWO-XGBoost model, and this gap is most pronounced at higher dry densities. This shows that Grey Wolf Optimization can have a slight edge against Particle Swarm Optimization in pinpointing flattened intervention in increased compaction. While the CNN model gives reliable prediction results; it demonstrates slightly more variation from practical data when compared with the GWO-XGBoost and the PSO-XGBoost approaches in some cases. The comparative analysis of both algorithms supposes the efficiency and interpretability of the models as the tools permits the predictability of the bentonite maximum a swelling pressure in geotechnical engineering applications and the GWO-XGBoost model takes a slight advantage in the predictive accuracy.

The superior performance of the GWO-XGBoost model can be attributed to the effective hyperparameter tuning achieved through the Grey Wolf Optimization algorithm. GWO efficiently explores the hyperparameter space by mimicking the social hierarchy and hunting behavior of grey wolves, enabling the identification of optimal hyperparameter values that enhance the predictive accuracy of the XGBoost model^87^. In comparison, the Particle Swarm Optimization algorithm, employed in the PSO-XGBoost model, optimizes hyperparameters by simulating the flocking behavior of birds or fish^79^. While both GWO and PSO are metaheuristic optimization techniques, GWO often demonstrates faster convergence and higher solution accuracy due to its ability to balance exploration and exploitation of the search space^87^. The computational complexity of GWO is O(t × n × d), where t is the maximum number of iterations, n is the number of search agents (wolves), and d is the dimensionality of the search space. Similarly, the computational complexity of PSO is O(t × n × d), with n representing the number of particles. Although the computational complexities of GWO and PSO are similar when integrated with XGBoost, the GWO-XGBoost model's faster convergence and higher accuracy make it more efficient and scalable for predicting the swelling pressure of bentonite-based materials in real-world applications.

### Model applicability and practical considerations

This further emphasizes the applicability of the XGBoost-GWO model in real-world scenarios. The data inputs of the designs suggested, including initial water content, dry density, plastic limit, liquid limit, and montmorillonite content, are typically determined through laboratory testing. With respect to changes in these parameters, the model faithfully reproduces the expected patterns across a broad range of bentonite kinds and combination combinations.

The comparative analysis presented in Table 8 demonstrates the superiority of the XGBoost-GWO model in predicting the maximum swelling pressure of bentonite and bentonite mixtures. The model's ability to handle complex relationships among input features, its robustness to outliers and noise, and the incorporation of a penalty term in the loss function set it apart from other approaches^3,107^. While the XGBoost-GWO model may be computationally intensive and require careful hyperparameter tuning^56,87^, its performance and reliability outweigh these limitations.

**Table 8:** Advantages and disadvantages of the machine learning models used in predicting bentonite swelling pressure.

Model	Advantages	Disadvantages
XGBoost-GWO	Handles complex relationships and interactions among input features^3^ Robust to outliers and noise in the data^107^ Incorporates a penalty term in the loss function to prevent unrealistic predictions^3^	Computationally intensive, especially with large datasets^56^ Requires careful tuning of hyperparameters to achieve optimal performance^87^
XGBoost-PSO	Efficiently explores the search space to find optimal hyperparameters^79^ Adapts the loss function to discourage predictions below a critical threshold^3^	May converge prematurely to suboptimal solutions^30,80^ Sensitive to the choice of hyperparameters and initialization^81^
CNN	Automatically extracts relevant features from input data^101^ Captures spatial and hierarchical patterns in the data^100^ Performs well with limited dataset sizes^25^	Requires a large amount of labeled training data for optimal performance^92^ Computationally expensive, especially with deep architectures^61^
LSTM-NN	Models long-term dependencies and sequential patterns in the data^64^ Handles vanishing and exploding gradient problems^65^ Applicable to regression tasks with well-defined input features^31^	Computationally intensive, especially with long sequences^104^ Requires careful initialization and regularization to prevent overfitting^108^
RBFNN	Faster learning capacity compared to other neural networks^70,71^ Simpler network architecture and easier to train^99^ Tolerant to errors in input variables^103^	Sensitive to the choice of basis function and its parameters^72^ May struggle with high-dimensional input spaces^75^ Requires careful selection of centers and widths^109^

In addition to the comparative analysis and novelty of our work, it is essential to consider the practical aspects of deploying and integrating the proposed machine learning models in real-world geotechnical engineering projects. When deploying the proposed machine learning models in real-world geotechnical engineering projects, several considerations should be taken into account. First, the quality and representativeness of the input data are crucial for the model's performance. It is essential to ensure that the data used for model training and testing cover a wide range of soil types, bentonite mixtures, and environmental conditions representative of the project site. Second, the model's predictive accuracy and robustness should be thoroughly evaluated using appropriate validation techniques, such as k-fold cross-validation and testing on independent datasets. Third, the model's limitations and uncertainties should be clearly communicated to the project stakeholders, and the results should be interpreted in conjunction with expert knowledge and engineering judgment. Finally, the model's performance should be continuously monitored and updated as new data becomes available during the project's lifetime to ensure its ongoing reliability and applicability.

To maximize the utility and impact of the proposed machine learning models, it is important to seamlessly integrate them into existing geotechnical analysis workflows. One approach is to develop user-friendly software tools or plugins that encapsulate the trained models and can be easily integrated into commonly used geotechnical software packages. These tools should allow users to input relevant soil properties and environmental conditions and provide reliable predictions of the maximum swelling pressure of bentonite and bentonite mixtures. Another approach is to establish a centralized database system that stores the input data, model parameters, and prediction results, facilitating data sharing and collaboration among geotechnical engineers and researchers. This database system can also serve as a platform for continuous model updates and improvements as new data becomes available. Additionally, it is crucial to provide comprehensive documentation and training materials to help geotechnical engineers understand the model's capabilities, limitations, and proper usage guidelines. By integrating these machine learning models into existing workflows and providing the necessary support and resources, their adoption and impact in geotechnical engineering practice can be greatly enhanced.

In addition to the practical aspects of model deployment and integration, it is crucial to consider the scalability and adaptability of the proposed machine learning models for large-scale applications. The scalability and adaptability of the proposed machine learning models are crucial factors to consider when applying them to large-scale geotechnical engineering projects. In this study, we have taken several steps to ensure the scalability and adaptability of the models. First, the use of a diverse dataset comprising 305 experimental data points from various sources, covering a wide range of bentonite types, mixtures, and testing conditions, helps to ensure the models' robustness and generalizability. Second, the application of k-fold cross-validation during the model training process helps to assess the models' performance on unseen data and reduces the risk of overfitting, thereby improving their adaptability to different datasets. Third, the feature importance analysis conducted for the GWO-XGBoost model (Fig. 6) provides insights into the relative contribution of each input parameter, allowing for the identification of the most influential factors affecting the swelling pressure of bentonite. This information can guide the selection of relevant input parameters when adapting the models to different datasets or conditions. Finally, the use of optimization algorithms, such as GWO and PSO, for hyperparameter tuning enhances the models' adaptability by automatically searching for the best combination of hyperparameters that maximize the models' performance on a given dataset.

Scaling up machine learning models for large-scale applications poses several challenges that need to be addressed. One of the key challenges is the computational complexity associated with training and deploying large-scale models. As the size of the dataset and the complexity of the model increase, the computational resources required for training and inference can become significant. To overcome this challenge, strategies such as distributed computing, parallel processing, and the use of cloud-based platforms can be employed to harness the power of multiple computing nodes and accelerate the model training and deployment process. Another challenge is the potential presence of data inconsistencies, noise, and outliers in large-scale datasets, which can adversely affect the models' performance. Robust data preprocessing techniques, such as outlier detection and removal, feature scaling, and data normalization, can help to mitigate these issues and improve the models' resilience to data quality problems. Additionally, the interpretability and explainability of large-scale machine learning models can become more challenging as the model complexity increases. The use of techniques such as feature importance analysis, as demonstrated in this study (Fig. 6), can help to provide insights into the model's decision-making process and enhance its interpretability. Furthermore, the development of user-friendly interfaces and visualization tools can facilitate the communication of model results to stakeholders and decision-makers.

The novelty of our work lies in the comprehensive evaluation of various machine learning models, including cutting-edge techniques like CNN and LSTM, and the development of a robust framework for estimating the swelling pressure of bentonite under diverse conditions. By incorporating a wide range of input features and providing detailed implementation information, our study addresses the limitations of existing research^16,31^ and contributes to the advancement of accurate and reliable models for predicting the behavior of bentonite-based materials in geotechnical engineering applications. The insights gained from our comparative analysis and the superior performance of the XGBoost-GWO model pave the way for more efficient and effective design and analysis of barrier systems, ultimately enhancing the safety and sustainability of various geotechnical projects.

**Figure 11:**
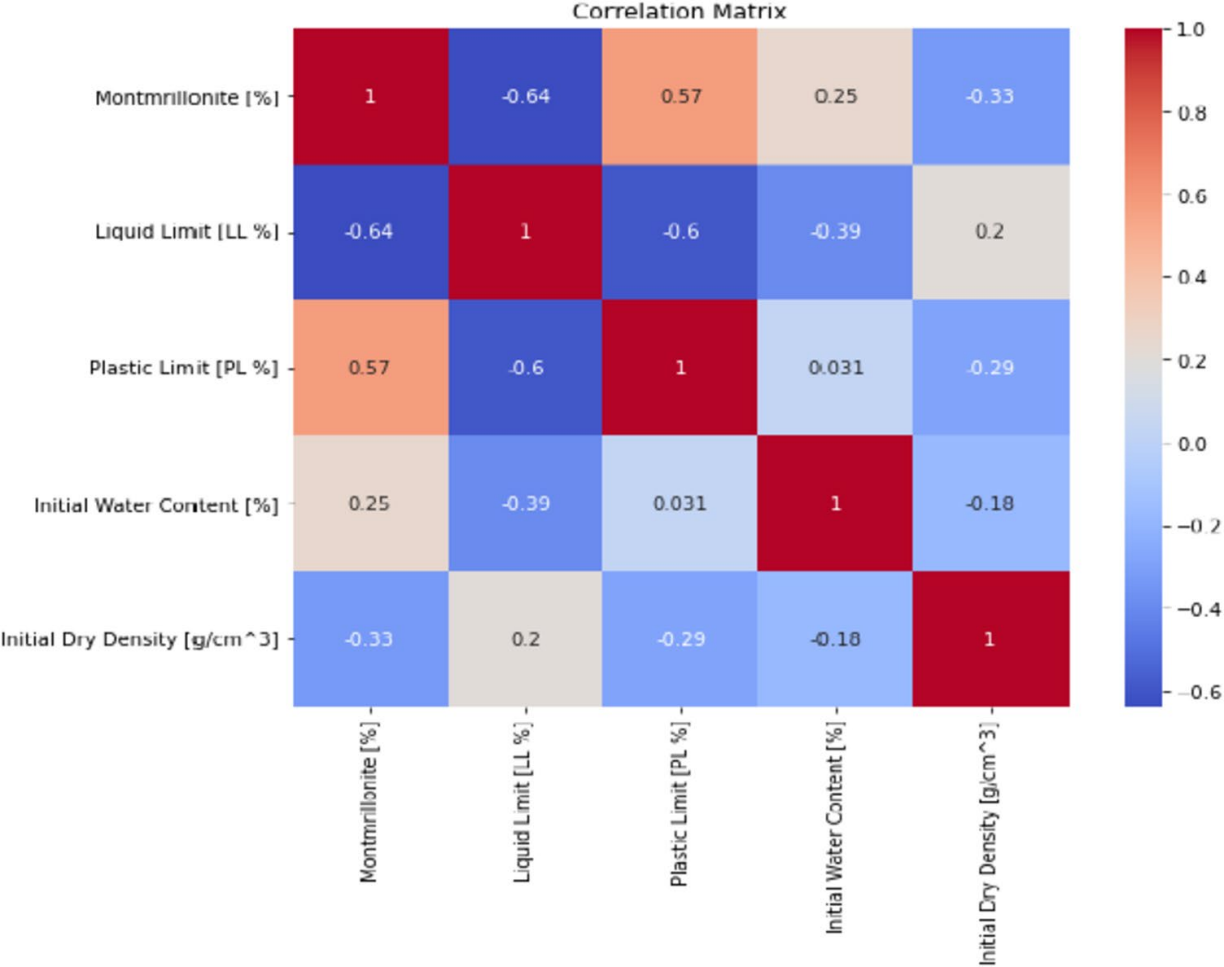
Relative Correlation heatmap.

The correlation matrix heatmap presented in Fig. 11 is a visual representation of the pairwise correlations between variables. The color scheme, ranging from shades of blue for negative correlations to shades of red for positive correlations, enables an intuitive understanding of the relationships among the variables. Darker hues signify stronger correlations in either direction. The heatmap reveals that montmorillonite content has a strong positive correlation (1.0) with itself, as expected. It also shows a moderately strong negative correlation (− 0.64) with the liquid limit, indicating that as the montmorillonite content increases, the liquid limit tends to decrease, and vice versa.

The plastic limit exhibits a weak positive correlation (0.57) with montmorillonite content, suggesting a slight tendency for the plastic limit to increase as the montmorillonite content increases. The plastic limit also has a moderate negative correlation (− 0.6) with the liquid limit, implying that higher values of the liquid limit are associated with lower values of the plastic limit. The initial water content shows a very weak positive correlation (0.031) with the plastic limit and a weak negative correlation (− 0.39) with the liquid limit, indicating little to no linear relationship between the initial water content and the plastic limit, and a slight inverse relationship with the liquid limit. The initial dry density has weak correlations with all other variables, ranging from − 0.33 with montmorillonite content to 0.2 with the liquid limit, − 0.29 with the plastic limit, and − 0.18 with the initial water content. These values suggest relatively weak associations between the initial dry density and the other variables. In summary, the heatmap reveals that montmorillonite content and liquid limit have the strongest correlation among all pairs, with a moderately strong negative relationship (− 0.64). The plastic limit shows a weak positive correlation with montmorillonite content (0.57) and a moderate negative correlation with the liquid limit (− 0.6). The initial water content and initial dry density have minimal impact on the overall correlation structure, with weak correlations with the other variables.

The comparative analysis with previous literature, as illustrated by the bar chart comparing the R-squared values of the proposed models with those of Schanz and Al-Badran^12^ and Komine and Ogata^18^ highlights how effectively the XGBoost-GWO model estimates the highest swelling pressure of materials made of bentonite. Subsequent investigations may concentrate on broadening the collection of data to comprise a more extensive range of soil kinds, mixing arrangements, and environmental circumstances, which might improve the models' resilience and suitability. Additionally, researching other hybridization strategies or machine learning techniques could open the door to the creation of models with improved or equivalent prediction completion.

### Statistical significance and overfitting

In our comparative analysis of the proposed machine learning models (XGBoost-GWO, XGBoost-PSO, RBFNN, LSTM-NN, and CNN), we evaluated their performance using various metrics such as R^2^, MSE, and RMSE, as presented in Tables 4, 6, and 7. These metrics provided valuable insights into the models' relative performance, with the XGBoost-GWO model consistently outperforming the other models. However, we acknowledge that our current study did not include specific statistical tests to assess the significance of the observed performance differences. In future work, it would be beneficial to conduct appropriate statistical tests, such as paired t-tests or Wilcoxon signed-rank tests, to quantify the statistical significance of these differences and provide a more rigorous basis for comparing the models.

Regarding potential overfitting, we employed several strategies to mitigate this risk. As mentioned in Section "Model Development", we used a separate testing dataset (20% of the total data) to assess the models' performance on unseen data. The results presented in Figs. 3, 7, and 8 demonstrate that the models, particularly the XGBoost-GWO model, achieved good performance on both the training and testing datasets, suggesting a reduced risk of overfitting. Furthermore, the incorporation of regularization techniques, such as the penalty term in the XGBoost models' loss function (Eqs. 34 and 35) and the use of early stopping and dropout layers in the neural network models (Section "Model Hyperparameters"), helped to discourage overly complex and overfit solutions.

To further assess the models’ generalization capabilities and potential overfitting, future research could explore the use of more advanced cross-validation techniques, such as nested k-fold cross-validation, and analyze the models' residuals for any systematic patterns indicative of overfitting. Additionally, the use of learning curves and other diagnostic tools could provide further insights into the models' performance and help identify any potential issues related to overfitting or underfitting.

In summary, while our current study did not include specific statistical tests for assessing performance differences or detailed analyses of overfitting, the employed evaluation metrics and regularization techniques suggest that the XGBoost-GWO model demonstrates superior performance and reduced overfitting risk compared to the other models. Future work should focus on conducting more rigorous statistical comparisons and exploring additional techniques for assessing and mitigating overfitting to further enhance the reliability and generalizability of the proposed models in the geotechnical context of predicting bentonite swelling pressure.

### Implications and contributions

Building upon the comprehensive comparative analysis and the superior performance of the GWO-XGBoost model, the current study significantly advances our understanding of bentonite swelling behavior by demonstrating the model's ability to accurately predict the maximum swelling pressure of bentonite-based materials. The model’s ability to accurately capture the complex relationships between various soil properties and swelling pressure, as evidenced by the high R-squared values and low error metrics (Fig. 3, Table 4), has important implications for the design and safety assessment of deep geological repositories. By providing a reliable tool for estimating swelling pressure under diverse conditions, the GWO-XGBoost model can help optimize the design of bentonite-based buffer and backfill systems, ensuring their long-term stability and performance. Moreover, the use of machine learning models, such as the GWO-XGBoost, offers significant environmental and economic benefits compared to traditional methods. These models can reduce the need for extensive laboratory testing, saving time, and resources, and minimizing the environmental impact associated with such tests. Furthermore, by enabling more accurate predictions of swelling pressure, the GWO-XGBoost model can help prevent over-design or under-design of repository components, thereby optimizing material usage and reducing construction costs. The feature importance analysis (Fig. 6) also provides valuable insights into the relative influence of different soil properties on swelling pressure, guiding future research efforts and aiding in the development of targeted interventions to mitigate excessive swelling. Overall, the findings of this study contribute to a more comprehensive understanding of bentonite swelling behavior and offer a powerful tool for enhancing the safety, efficiency, and sustainability of deep geological repository systems.

## Conclusions

In this study, we developed and evaluated a novel machine learning approach using the XGBoost algorithm optimized with Grey Wolf Optimization (GWO) and Particle Swarm Optimization (PSO) techniques to accurately predict the maximum swelling pressure of bentonite and bentonite mixtures. The performance of the XGBoost-based models was compared with that of advanced neural network architectures, namely Radial Basis Function Neural Network (RBFNN), Long Short-Term Memory Neural Network (LSTM-NN), and Convolutional Neural Network (CNN), all optimized using the Adam optimizer.

The superior performance of the XGBoost-GWO model can be attributed to its ability to handle complex relationships among input features, its robustness to outliers and noise, and the incorporation of a penalty term in the loss function. The comparative analysis with experimental data from various studies demonstrated the reliability and adaptability of the developed models in predicting the swelling pressure of bentonite-based materials under diverse conditions.

The insights gained from this research pave the way for the development of more accurate and efficient predictive tools in geotechnical engineering applications, particularly in the design and analysis of barrier systems for radioactive waste disposal. Future research directions could focus on extending the application of the XGBoost-GWO model to other geotechnical engineering problems, integrating advanced feature selection techniques, and exploring hybrid machine learning approaches to further enhance the predictive capabilities of the models.

We acknowledge that our dataset, consisting of 305 data points, is relatively small compared to the large datasets often used in deep learning applications. However, it is important to note that the required dataset size depends on the complexity of the problem and the model architecture. In our study, we have carefully designed the CNN and LSTM models to avoid overfitting by employing appropriate regularization techniques and cross-validation. Moreover, we have ensured that the models are trained until convergence, and we have reported the results based on multiple runs with different random seeds to account for the potential variability in performance. While a larger dataset could potentially improve the model’s performance and generalization ability, we believe that our current dataset is sufficient to demonstrate the effectiveness of the proposed approach in predicting the swelling pressure of bentonite. Nevertheless, we acknowledge that the model’s performance might be further enhanced by expanding the dataset in future studies, and we encourage other researchers to build upon our work by incorporating additional experimental data.

To further enhance the predictive capabilities of the proposed models, future research should focus on addressing the limitations highlighted in this study. The GWO-XGBoost model could be improved and expanded by incorporating additional variables that influence the swelling behavior of bentonite, such as mineralogical composition, pore water chemistry, and temperature. Moreover, the model's applicability to different types of bentonite and bentonite-based mixtures should be investigated to enhance its robustness and versatility. This could be achieved by incorporating a wider range of bentonite samples from various geological sources into the training dataset. Additionally, the integration of other advanced machine learning techniques, such as deep learning algorithms, could be explored to capture more complex and nonlinear relationships between swelling pressure and the relevant input parameters. By addressing these limitations and expanding the model's capabilities, we can develop a more comprehensive and reliable tool for predicting the swelling pressure of bentonite under diverse environmental conditions and for various geotechnical applications.

## Data Availability

The datasets used and/or analysed during the current study available from the corresponding author on reasonable request.
